# Integrated multi-omics analysis of Huntington disease identifies pathways that modulate protein aggregation

**DOI:** 10.1242/dmm.049492

**Published:** 2022-10-31

**Authors:** Sai S. Pradhan, Sai M. Thota, Saiswaroop Rajaratnam, Sai K. S. Bhagavatham, Sujith K. Pulukool, Sriram Rathnakumar, Kanikaram S. Phalguna, Rajesh B. Dandamudi, Ashish Pargaonkar, Prasanth Joseph, E. V. Joshy, Venketesh Sivaramakrishnan

**Affiliations:** ^1^ Disease Biology Lab, Department of Biosciences, Sri Sathya Sai Institute of Higher Learning, Prasanthi Nilayam, Anantapur, Andhra Pradesh, India 515134; ^2^ Department of Chemistry, Sri Sathya Sai Institute of Higher Learning, Prasanthi Nilayam, Anantapur, Andhra Pradesh 515 134, India; ^3^ Application Division, Agilent Technologies Ltd., Bengaluru 560048, India; ^4^ Department of Neurology, Sri Sathya Sai Institute of Higher Medical Sciences, Whitefield, Bengaluru, Karnataka 560066, India

**Keywords:** Huntington disease, Metabolomics, Multi-omics analysis, Neurodegenerative disease, Protein aggregation, HD yeast model

## Abstract

Huntington disease (HD) is a neurodegenerative disease associated with polyglutamine expansion in the protein huntingtin (HTT). Although the length of the polyglutamine repeat correlates with age at disease onset and severity, psychological, cognitive and behavioral complications point to the existence of disease modifiers. Mitochondrial dysfunction and metabolic deregulation are both associated with the HD but, despite multi-omics characterization of patients and model systems, their mechanisms have remained elusive. Systems analysis of multi-omics data and its validation by using a yeast model could help to elucidate pathways that modulate protein aggregation. Metabolomics analysis of HD patients and of a yeast model of HD was, therefore, carried out. Our analysis showed a considerable overlap of deregulated metabolic pathways. Further, the multi-omics analysis showed deregulated pathways common in human, mice and yeast model systems, and those that are unique to them. The deregulated pathways include metabolic pathways of various amino acids, glutathione metabolism, longevity, autophagy and mitophagy. The addition of certain metabolites as well as gene knockouts targeting the deregulated metabolic and autophagy pathways in the yeast model system showed that these pathways do modulate protein aggregation. Taken together, our results showed that the modulation of deregulated pathways influences protein aggregation in HD, and has implications for progression and prognosis.

This article has an associated First Person interview with the first author of the paper.

## Teaser

**Summary:** Metabolomic analysis of Huntington disease (HD) patients and of a HD yeast model identified deregulated metabolic pathways. We used the yeast model to investigate their role in the aggregation of huntingtin protein and their implications in HD.

## INTRODUCTION

Neurodegenerative diseases, such as Huntington disease (HD), Alzheimer disease (AD), Parkinson disease (PD) and amyotrophic lateral sclerosis (ALS) are incurable. These diseases are associated with the formation of protein aggregates that eventually leads to neuronal cell death (Wolfe and Cyr, 2011). Despite pooling considerable efforts, a cure has largely been elusive. Clinical trials that used antibodies against protein aggregates or their soluble oligomers have failed (Mehta et al., 2017; Kingwell, 2021; Denis et al., 2019), indicating the gaps that exist in the understanding of the biology of protein aggregating diseases. HD, which affects the central nervous system (CNS), is caused by cytosine-adenine-guanine (CAG) repeats in the huntingtin gene (*HTT*), resulting in an expanded polyQ stretch within the HTT protein, leading to neurodegeneration (MacDonald et al., 1993; Trottier et al., 1995). Although the disease exhibits a mid-life onset, a juvenile form of HD has also been reported (Nance and Myers, 2001) and the polyQ length correlates with the age of disease onset (Snell et al., 1993; Andrew et al., 1993; Duyao et al., 1993; Chen et al., 2002). HD patients develop different complications, such as depression, behavioral and cognitive dysfunction, indicating other factors that can influence the course of disease (Roos, 2010).

Genetic modifiers of HD with variable penetrance have been reported [Gusella and MacDonald, 2009; Gusella et al., 2014; Genetic Modifiers of Huntington's Disease (GeM-HD) Consortium, 2015, 2019]. Previous studies have shown mitochondrial dysfunction and SNPs in genes involved in the mitochondrial electron transport chain [Genetic Modifiers of Huntington's Disease (GeM-HD) Consortium, 2015; Carmo et al., 2018]. HD patient lymphocytes show significant higher mutation frequency of mitochondrial DNA (mtDNA) and reduced expression of factors that are essential for mtDNA replication, repair or degradation (Petersen et al., 2014). Further, mitochondrial fission and fusion are important events regulating the metabolic state of mitochondria, which are deregulated in HD (Reddy, 2014). Mitophagy helps to maintain the health and quality of mitochondria (Yang et al., 2018). Mitochondrial dysfunction in HD is associated with elevated levels of reactive oxygen species (ROS), resulting in oxidative stress, which – in turn – is implicated in neuronal cell death (Johri and Beal, 2012; Paul and Snyder, 2019; Kumar and Ratan, 2016).

In HD patients, pyruvate dehydrogenase (PDH) activity is impaired, which suggests it has a role in the initiation or propagation of disease (Sorbi et al., 1983). MRI and PET scan studies have shown differences in the metabolic profile and glucose utilization, respectively, in brains of HD patients compared with normal brain (Niccolini and Politis, 2014; Ma and Eidelberg, 2007). Recent findings suggest involvement of the Warburg and/or inverse Warburg effect – i.e. upregulation of glucose consumption concomitant with lactate production and/or glucose production concomitant with lactate consumption – in diseases that feature abnormal protein aggregates (Demetrius et al., 2015). HD – which leads to neurodegeneration – has been shown to exhibit changes in levels of various metabolites (den Bogaard et al., 2011; Gu et al., 1996; Tabrizi et al., 2000; Turner et al., 2007). By using cell line models, deregulation of the homocysteine and tryptophan-kynurenine pathway has been implicated in the disease (Paul et al., 2014; Yang et al., 2017). Lipid metabolism has also been shown to have a role in the disease (Adibhatla and Hatcher, 2007). Overall, metabolic remodeling has been associated with HD (Rosenstock et al., 2010; Feigin et al., 2007), which might have potential implications for the disease.

The metabolic status of the cells has been shown to modulate the autophagy pathway (Lahiri et al., 2019). Aggregates of mutant HTT are cleared via the autophagy pathway, a mechanism that is impaired in HD patients (Mputhia et al., 2019; Martinez-Vicente et al., 2010). Consistent with these observations, mTOR – which inhibits autophagy – is activated in HD patients (Ravikumar et al., 2004), and impaired autophagy, deregulated signaling and metabolic pathways, as well as mitochondrial dysfunction, might modulate the formation of protein aggregates in HD.

Transcriptomics analyses of the affected region in brains obtained from HD patients or model systems showed deregulation of signaling and metabolic pathways (Durrenberger et al., 2012; Hodges et al., 2008). Moreover, proteomics and metabolomics analyses of HD patients reiterated the role of deregulated metabolic pathways in this disease. Some studies have shown the efficacy of lipoic acid, dichloroacetate or IDO1 and IDO2 inhibitors by achieving a favorable prognosis in HD patients (Andreassen et al., 2001a; Andreassen et al., 2001b; Boros et al., 2019). The standard of care has largely focused on suppressing symptoms in HD patients (McColgan and Tabrizi, 2018). Although many omics studies have shown an association of deregulated pathways with the diseases, their role in protein aggregation and toxicity of mutant HTT is not well appreciated. Hence, a system-level understanding of HD through integrative analysis of omics and other datasets will not only help to understand the biology but will also help to identify potential biomarkers associated with disease progression and prognosis, as well as therapeutic targets to manage the disease.

Yeast has been used as a model system to study the role of pathways in many diseases (Bolotin-Fukuhara et al., 2010; Mohammadi et al., 2015). It has also emerged as a model system to study protein aggregates that are induced in neurodegenerative diseases (Tauber et al., 2011). Studies in yeast models of HD have demonstrated the role of cellular chaperones in modulating protein aggregates (Meriin et al., 2002). Autophagy network and proteasome has been conceived in the degradation of protein aggregates in HD (Nedelsky et al., 2008). Deregulation of the tryptophan-kynurenine pathway has been demonstrated in the yeast model of HD (Stone et al., 2012). Genetic screening discovered genes that modulate the toxicity of HTT-polyQ in yeast model of HD (Meriin et al., 2002; Tauber et al., 2011; Serpionov et al., 2017). Overall, yeast has emerged as a model to investigate several deregulated pathways regarding HTT aggregation in HD. With this wealth of multi-omics datasets and established model systems, HD is amenable to integrated analysis. An integrated analysis followed by validation by using yeast as a model system will help to understand the role of deregulated pathways in protein aggregation and, hence, in disease process.

In this study, we metabolomically analyzed pre-symptomatic and symptomatic HD patients, individuals as familial controls as well as age- and sex-matched controls, and categorized the significantly different levels of metabolites into pathways. Furthermore, metabolomics analysis of the HD yeast model was carried out and the significantly different levels of metabolites were categorized into pathways. Comparative analysis of the metabolic pathways – obtained by analysis of GEO-based transcriptomics datasets as well as of proteomics and metabolomics datasets obtained from literature of patients and model systems (Table S1) – have been carried out with our datasets. We further validated the role of genes and metabolites from the deregulated pathways for their role in HTT protein aggregation by using the yeast model of HD. The pathway common to all the datasets (yeast, mouse and human), or those common between yeast and human or yeast and mouse were probed for their role in modulating protein aggregation. We also investigated the role of deregulated pathways observed only in human but conserved in yeast. Knockout of yeast orthologs corresponding to those genes associated with pathways deregulated in humans and mouse was used to understand the ability of these pathways to modulate protein aggregation. Our results are discussed considering the role of different deregulated pathways in modulating protein aggregation and their potential implications for biomarkers or therapeutic targets.

## RESULTS

### Metabolomics analysis of HD patients compared to that of familial, and age- and sex-matched controls shows deregulation of metabolic pathways

**Fig. 1.**
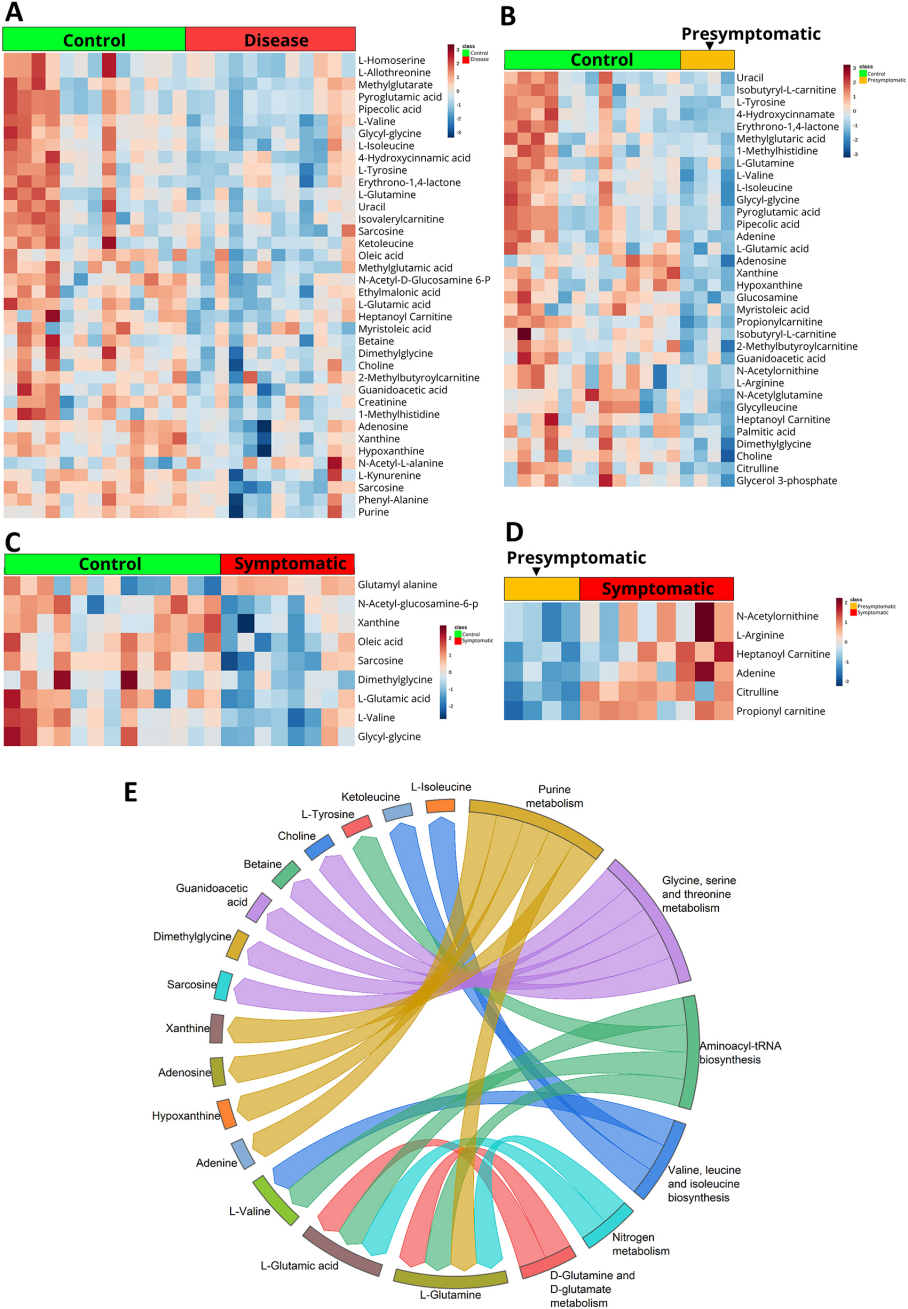
**Human serum sample metabolomics.** (A-D) Heatmaps representing the level of significantly altered metabolites at FDR≤0.25, control and disease (A), control and asymptomatic patients (B), control and symptomatic patients (C) and asymptomatic and symptomatic patients (D). (E) Plotted are the results of the metabolite set enrichment analysis (MSEA), showing the link between significantly altered metabolites and six metabolic pathways they impact on, when comparing HD patient and control samples. Pathways were considered to be significantly enriched at a false discovery rate (FDR) value of ≤0.25.

Metabolomics analysis of pre-symptomatic (*n*=5) and symptomatic (*n*=11) HD patients, as well as that of familial controls (*n*=11) and age- and sex-matched controls (*n*=4) were carried out. The patient cohort had been previously characterized considering the length of CAG repeats; outcome of multimodal magnetic resonance imaging (MRI), i.e. MRI, MRS, DTI, ADC scans; positron emission tomography (PET) scans and visual evoked potential (VEP) measurements (Thota et al., 2021). Briefly, serum samples were processed as described in Materials and Methods and 2 μl of the sample was used for liquid chromatography–mass spectrometry (LC–MS). Targeted metabolomics analysis was carried out for 165 metabolites, 89 of which were detected. The peak intensities of the detected metabolites were normalized to L-tryptophan used as the internal standard. Metabolites with a coefficient of variation (CV) ≤20% after normalization were chosen for further analysis. MetaboAnalyst 5.0 was used for all analyses. Of the 31 samples, which include controls, familial control, pre-symptomatic and symptomatic HD patients, four were outliers (one symptomatic, one pre-symptomatic and two familial controls) that were excluded from analysis. Details of the metabolite heatmap and the partial least squares discriminant analysis (PLS-DA) variant on all samples are shown in Figs S1 and S2. Similarly, Student's *t*-test performed for familial and age- and sex-matched controls as well as pre-symptomatic and symptomatic HD patients showed 38 significantly different levels of metabolites (Fig. 1A). PLS-DA clustered these samples into two groups, which are represented as a 2D score plot (Fig. S3). The significant metabolites are represented as a heatmap (Fig. 1A).

The metabolomics datasets were further analyzed for significantly different levels of metabolites between pre-symptomatic patients, and both familial and age- and sex-matched controls. Obtained were 34 significantly different levels of metabolites after analyzing with MetaboAnalyst 5.0; results are provided in a Heatmap (Fig. 1B). The PLS-DA 2D score plot clustered the samples into two groups and is represented in Fig. S4. Analysis of metabolomics datasets for significantly different levels of metabolites in symptomatic patients as well as familial and age- and sex-matched controls yielded nine metabolites (Fig. 1C) – with the PLS-DA 2D score plot represented in Fig. S5, whereas significantly different levels of metabolites between pre-symptomatic and symptomatic patients yielded six metabolites (Fig. 1D). The PLS-DA 2D score plot is provided in Fig. S6.

Pathway analysis using Metabolite Set Enrichment Analysis (MSEA) was carried out by comparing HD patient and control samples and using the significantly different metabolites. Our analysis categorized these significantly different metabolites into six pathways – three metabolic pathways of amino acids, i.e. (i) glycine, serine and threonine metabolism; (ii) valine, leucine and isoleucine biosynthesis; (iii) D-glutamine and D-glutamate metabolism, as well of (iv) purine metabolism, (v) aminoacyl-tRNA biosynthesis and (vi) nitrogen metabolism. Results are provided as a chord diagram, which provides details of pathways and associated metabolites (Fig. 1E).

### Metabolomics analysis of the HD yeast model shows a deregulation of metabolic pathways

The yeast model of HD has been previously used to probe the roles of chaperones and various genes in the formation of HTT protein aggregates (Hofer et al., 2018; Krobitsch and Lindquist, 2000). Here, we used yeast expressing the N-terminus of HTT coupled to the CAG triplet code encoding glutamine, linked to EGFP (N-Terminal-HTT-polyQ-EGFP) from the plasmid p426 GPD (i.e. p426 25Q GPD, p426 46Q GPD, p426 72Q GPD and p426 103Q GPD, procured from Addgene; see Materials and Methods for further details). A polyQ length of 25Q was considered normal, whereas lengths of 46Q, 72Q and 103Q represent HD-causing mutants. The yeast sets are specified by the polyQ length and, hereafter, are referred to as 25Q, 46Q, 72Q and 103Q. Diffuse fluorescence foci were produced by 25Q, whereas 46Q produced very small fluorescent foci, representing protein aggregate in some cells; however, the majority of cells exhibited diffuse fluorescence. The provided figures are representative of our observations (Fig. S7a). 72Q and 103Q produced big fluorescent foci that represent HTT protein aggregation. Further, filter retardation assay was carried out to probe the protein aggregate formation and an anti-GFP antibody was used to probe the different polyQ proteins, as described in Materials and Methods (Fig. S7b). Consistent with previous reports, the results of the filter retardation assay showed that 25Q exhibited little aggregate formation, whereas 46Q exhibited some, but less intense, HTT protein aggregation (Fig. S7b). 72Q and 103Q, however, exhibited intense HTT protein aggregation formation (Fig. S7b). These results are in concordance with those obtained by fluorescence imaging (Fig. S7a). The yeast growth assay was performed to evaluate if the polyQ showed changes in growth characteristics. The results of our dilution assay did not show significant changes in growth characteristics (Fig. S7c) or optical density of the culture (OD_600_=0.5 in all culture samples). Importantly, these observations are consistent with previous studies (Krobitsch and Lindquist, 2000).

Metabolomics analysis of the yeast model of HD was then carried out. The metabolomics data of 46Q, 72Q and 103Q were compared to that of 25Q,which served as control. Briefly, 8×10^6^ cells each were homogenized after spiking them with a mixture of labelled, i.e. L-tryptophan, pyruvate, glucose, and unlabeled, i.e. jasmonic acid, metabolites used as standards. The resulting homogenates were centrifuged and their supernatants passed through a 3 kDa cut-off filter. From the resulting filtrate, 2 μl were used for LC-MS. The LC-MS system was either operated in positive or negative ionization mode to produce and detect positive and negative ions, respectively. The total number of metabolites targeted in the positive ionization mode was 165, of which 109 were identified, whereas the total number of metabolites targeted in the negative ionization mode was 91, of which 49 were detected. All identified metabolites were normalized to the internal standards. As internal standards labelled L-tryptophan was used for positive and unlabeled jasmonic acid for negative mode. PLS-DA analysis for each of the disease mutations and the control sets clustered into different groups, which are provided as a 2D score plot (Figs S8-S13).

**Fig. 2.**
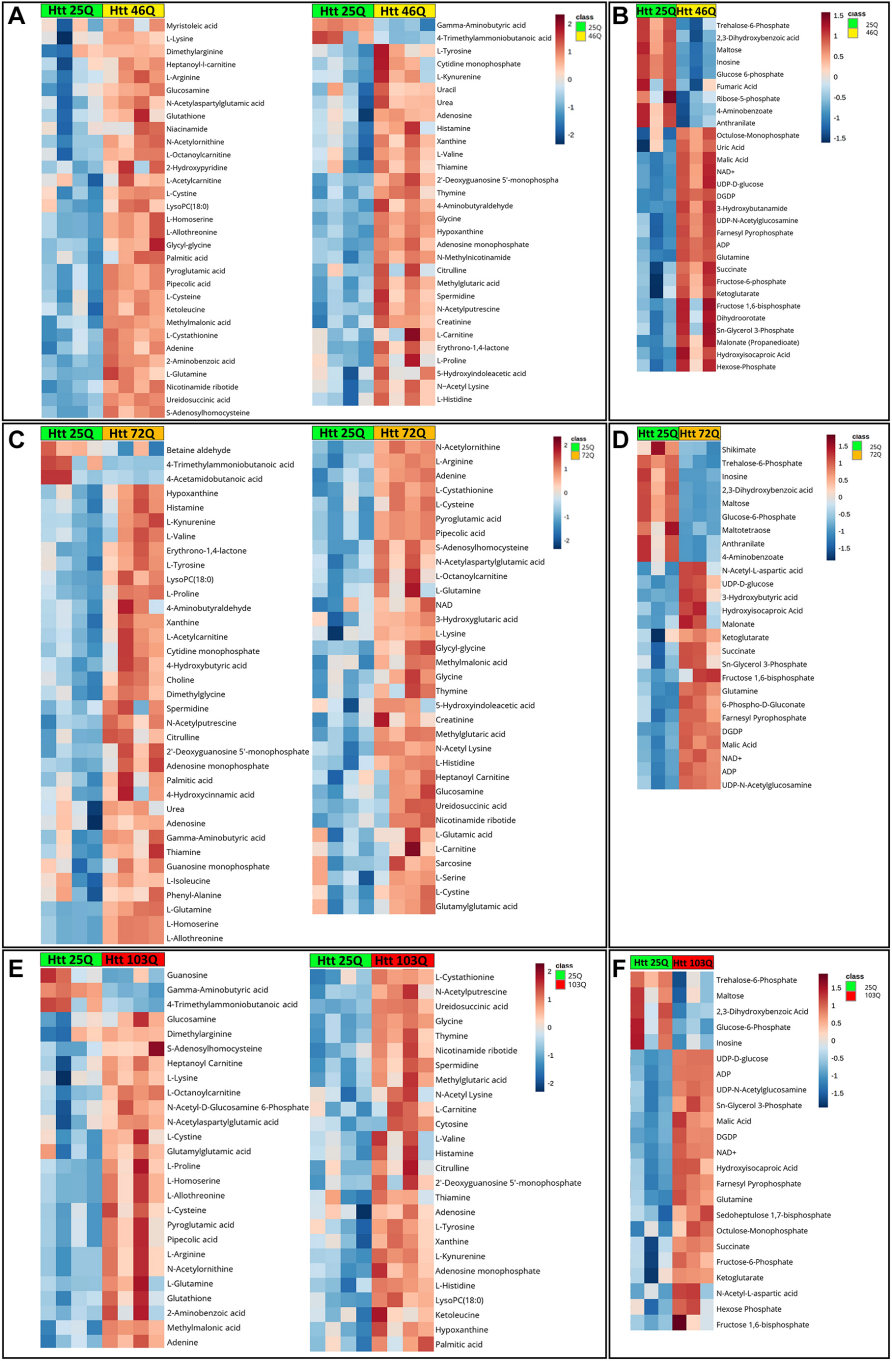
**Metabolomic study using an HD yeast model.** (A-F) Heat maps of targeted metabolomics in Htt yeast models 46Q, 72Q and 103Q compared to that of 25Q in positive and negative modes. Altered metabolites in 46Q compared to 25Q in positive (A) and negative (B) mode. Altered metabolites in 72Q compared to 25Q in positive (C) and negative mode (D). (E) Altered metabolites in 103Q compared to 25Q in positive (E) and negative (F) mode.

**Fig. 3.**
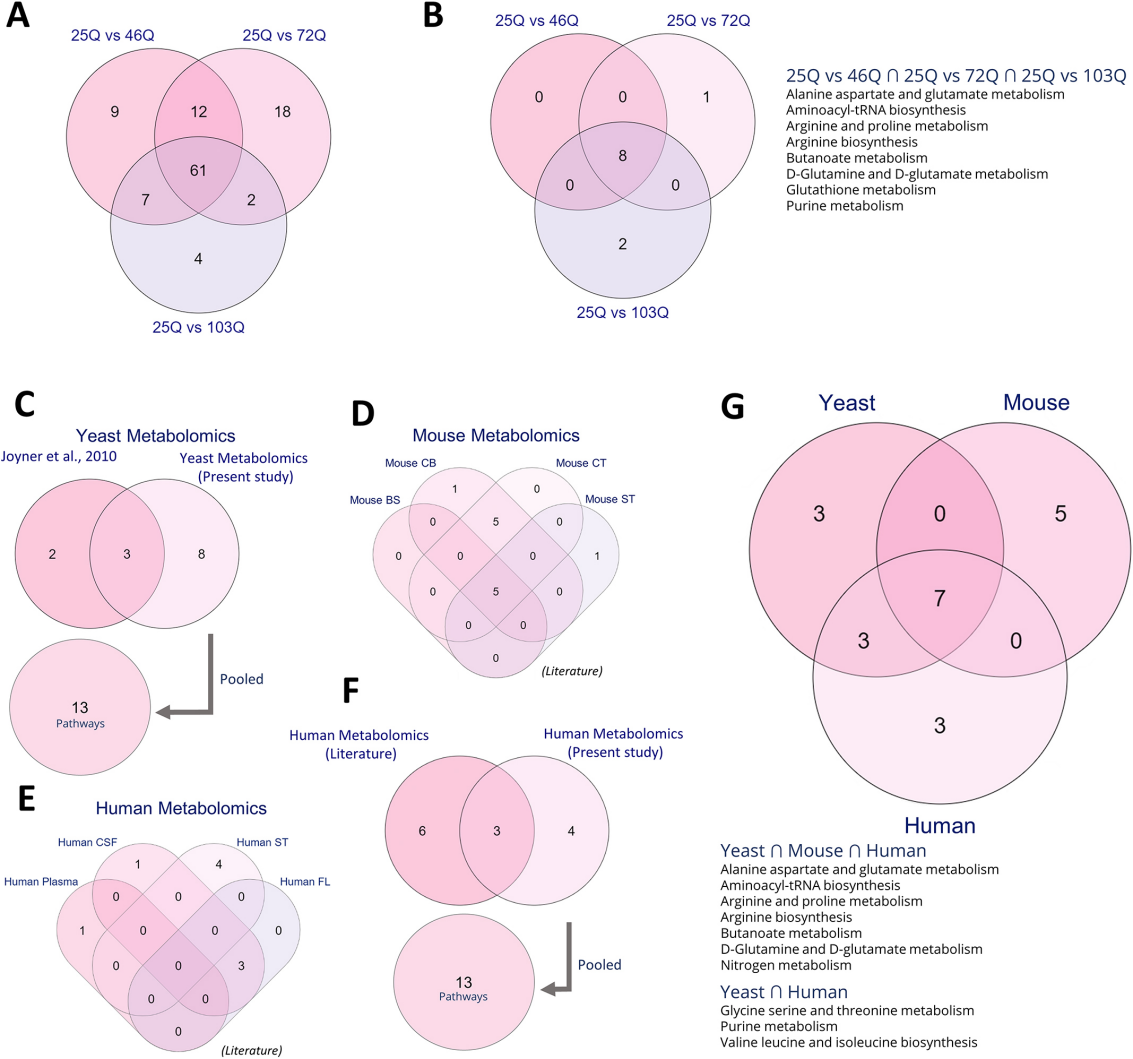
**Metabolite set enrichment analysis (MESA) and shared deregulated pathways.** (A) Yeast metabolomics (present study) show significantly different levels of metabolites in yeasts 46Q, 72Q and 103Q compared to 25Q. (B) MSEA showing shared pathways deregulated in 25Q vs 46Q, 25Q vs 72Q and 25Q vs 103Q. (C) Metabolomics pathway overlaps determined in our study (yeasts 25Q vs 46Q, 25Q vs 72Q and 25Q vs 103Q) and literature show pathways commonly deregulated in yeast 103Q compared to yeast 25Q. (D,E) Comparative analysis of significantly enriched metabolic pathways identified in a HD mouse model (D) and in HD patients (E). (F) Metabolomics of previously published deregulated metabolic pathways in human (literature) and deregulated pathways identified in our HD patient cohort (present study), showing an overlap of three deregulated pathways. (G) Plotted is the overlap of deregulated metabolic pathways identified in yeast and mouse HD models, and HD patients. BS, brain stem; CB, cerebellum; CSF, cerebrospinal fluid; CT, cortex; FL, frontal lobe; ST, striatum.

Analysis of 46Q compared with 25Q as control showed 61 significant metabolites in the positive mode and 29 in the negative mode (Fig. 2A,B). Analysis of 72Q compared with 25Q as control showed 68 significant metabolites in the positive and 26 in the negative mode (Fig. 2C,D). Analysis of 103Q compared with 25Q as control showed 52 significant metabolites in the positive and 23 in the negative mode (Fig. 2E,F). Considerable overlap of the identified significantly different levels of metabolites (61 metabolites) was observed between 46Q, 72Q and 103Q (Fig. 3A). The total number of pathways obtained from the significantly different levels of metabolites for 46Q, 72Q and 103Q was eight, nine and ten, respectively (Fig. 3B). Commonality analysis of metabolic pathways between 46Q, 72Q and 103Q conjured eight pathways (Fig. 3B). The common pathways include metabolism of amino acids (alanine, aspartate, glutamate, glutamine, arginine, proline), aminoacyl-tRNA biosynthesis, butanoate, glutathione and purine metabolism (Fig. 3B). The mutant sets 72Q and 103Q had one and two metabolic pathways, respectively, that are unique to them (Fig. 3B).

### Comparison of our human and yeast metabolomics datasets with those previously published shows shared, deregulated pathways

Metabolomics datasets analyzed include those from yeast and mouse striatum, cerebellum, cortex and brain stem (Fig. 3D), and from human blood plasma, cerebrospinal fluid (CSF), frontal lobe and striatum (Fig. 3E).

We compared the pathways obtained from our yeast metabolomics datasets with those from analysis of previously published datasets (Joyner et al., 2010). Our analysis shows three metabolic pathways that are shared between the yeast metabolic datasets (Joyner et al., 2010) (Fig. 3C). Four human metabolic datasets obtained from literature (Graham et al., 2016, 2018; Herman et al., 2019; Rosas et al., 2015) were analyzed for pathways, and commonality analysis was performed between them. In humans, overlap of three pathways was observed between two of the datasets (Fig. 3E). Furthermore, the pathways obtained from analysis of data from literature were pooled and compared with those obtained for the HD patient cohort in our study. Analysis showed three shared pathways between the pooled metabolic pathways in humans, i.e. pathways identified in previously published data and in our patient cohort (Fig. 3F). Similarly, four mouse metabolic datasets obtained from literature (Tsang et al., 2006) were analyzed for pathways and commonality, and results show five shared metabolic pathways between all mouse datasets, whereas another five were found to be shared between only two datasets (Fig. 3D). Details on pathways shared between mouse and human datasets are provided in Fig. S14.

In addition, we performed commonality analysis between pooled datasets of yeast (13 pathways), human (13 pathways) and mouse (12 pathways). Our analysis shows that seven metabolomics pathways were shared between all different datasets, whereas yeast and human datasets had three overlapping pathways between them. The common pathways that emerged between all three pooled datasets include aminoacyl-tRNA biosynthesis, metabolism of amino acids (alanine, aspartate, glutamate, glutamine, arginine and proline) and nitrogen metabolism (Fig. 3G).

**Fig. 4.**
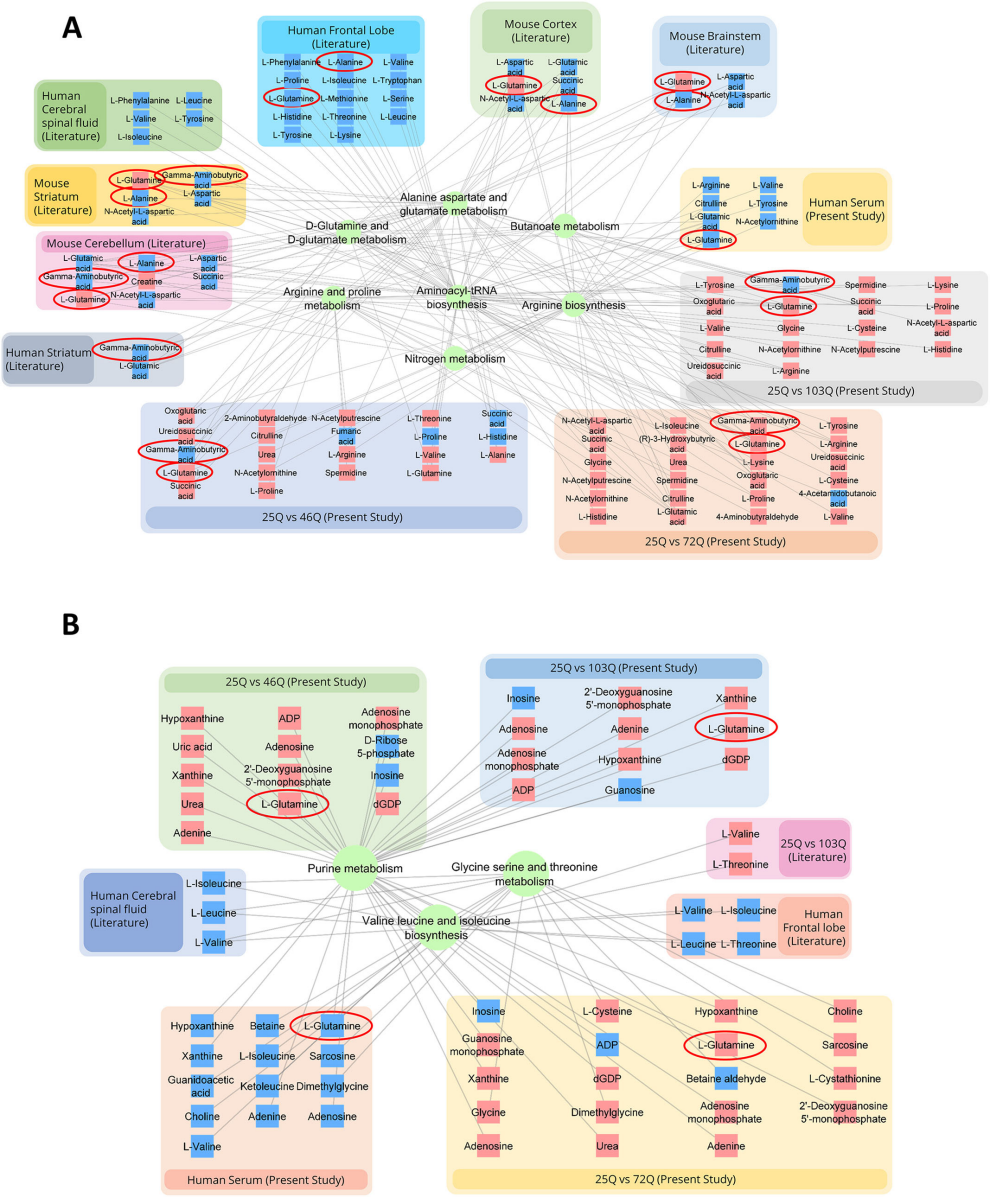
**Shared deregulated metabolics pathways.** (A) Network representation of deregulated pathways that are shared between yeast and mouse models of HD and HD patients. (B) Shared deregulated metabolic pathways showing yielded metabolites for respective datasets, unique to HD patients and HD yeast models. Blue partial underlays indicate downregulated metabolites, red partial underlays indicate upregulated metabolites. L-Glutamine, L-alanine and gamma aminobutyric acid metabolites are encircled.

Furthermore, we performed a commonality analysis for the shared pathways together with the associated metabolites between pooled datasets from human, mouse and yeast (Fig. 3G). Interestingly, although the pathways are similar, levels of the associated metabolites are different (Fig. 4). However, despite the differences, levels of alanine, glutamine and GABA were very similar between the analyzed dataset, except for those of glutamine in the human datasets (Fig. 4A,B).

### Transcriptomics analysis identified deregulated pathways between the yeast HD model and affected brain region of HD patients and the mouse HD model

Transcriptomics datasets were obtained from the GEO databases for yeast (Tauber et al., 2011), brain hemisphere (R6/1) (Hodges et al., 2008) and striatum [Mouse (CHL2)] of mouse (Kuhn et al., 2007), as well as the ventral head of the caudate nucleus (VHCN) (Durrenberger et al., 2012), peripheral blood samples (Borovecki et al., 2005) and iPSC-derived neuronal progenitor cells (NPCs) of HD patients (Xu et al., 2017). Details on transcriptomics datasets used for the study are provided in Table S1.

**Fig. 5.**
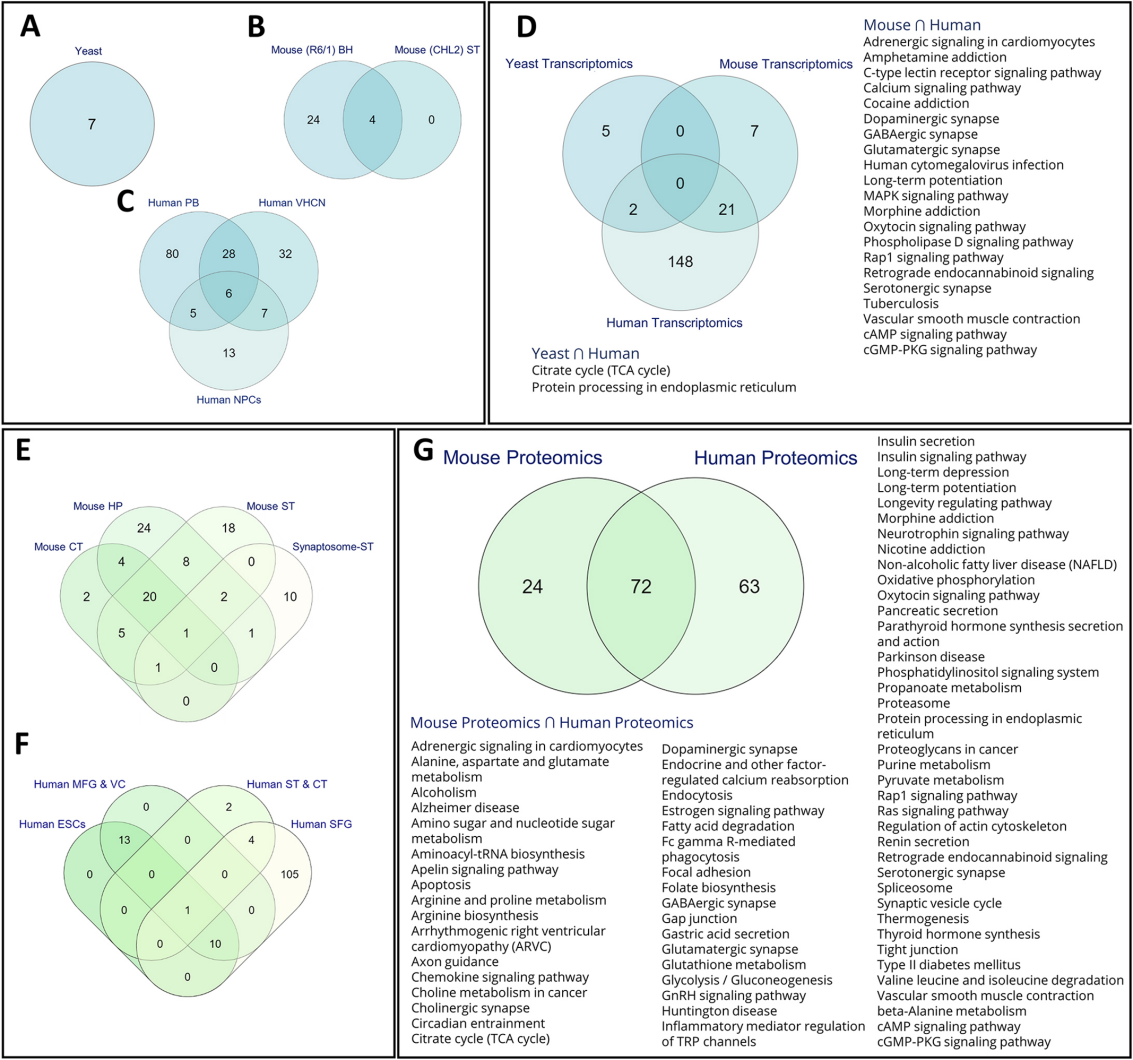
**Enrichment analysis – transcriptomics and proteomics.** (A-C) Functional enrichment analyses of differentially expressed genes in HD patients and yeast, mice model of HD. Venn diagrams show the number of pathways shared between different samples obtained from yeast (A), mice (B) or HD patients (C). (D) Venn diagram highlighting shared pathways in samples obtained from HD patients, and from yeast and mouse HD model systems. (E,F) Venn diagrams showing proteomics data analysis of shared deregulated pathways in samples obtained from HD mice models (E) and HD patients (F). (G) Venn diagram showing proteomics data of HD mouse models and human patients, highlighting shared deregulated pathways. BH, brain hemisphere; CT, cortex; HP, hippocampus; NPCs, neuronal progenitor cells; MFG & VC, middle frontal gyrus and visual cortex; PB, peripheral blood; SFG, superior frontal gyrus tissue; ST, striatum; ST & VC, striatum and visual cortex; VHCN, ventral head of the caudate nucleus.

Analysis of yeast transcriptomics data showed a total of seven pathways (Fig. 5A). The transcriptomics data from mouse brain hemisphere and mouse striatum showed overlap of four pathways (Fig. 5B). The human transcriptomics datasets showed overlap of six metabolic pathways between all three datasets (Fig. 5C). A total of five pathways were shared between human peripheral blood samples and human NPC, whereas a total of seven pathways were shared between human NPC and human VHCN, and 28 between human peripheral blood samples and human VHCN (see Fig. S15 for further details).

The pathways obtained by analysis of individual yeast, mouse and human transcriptomics datasets were combined and commonality pathway analysis between the different species was carried out. Yeast and human datasets showed two shared pathways, which include the tricarboxylic acid (TCA; also known as citric acid or Krebs) cycle and protein processing in the endoplasmic reticulum (Fig. 5D). The mouse and human datasets showed 21 shared pathways (Fig. 5D), including the Calcium-, C-type lectin-, oxytocin-, MAPK-, Rap1- and phospholipase D-signaling pathways, and the dopaminergic, serotonergic, GABAergic and glutamatergic pathways. (Fig. 5D).

We also separately performed pathway analysis of genes up- or downregulated in the transcriptomics datasets of yeast and mouse HD models, and HD patients (Figs S16-S21). For HD yeast datasets, we found that the enriched pathways obtained from upregulated genes include those belonging to fructose and mannose metabolism, TCA cycle, glutathione metabolism, pentose phosphate pathway (PPP) and protein processing in the endoplasmic reticulum, and also that the enriched pathways obtained from downregulated genes include those belonging to riboflavin metabolism and thiamine metabolism (Fig. S16). For HD mouse striatum datasets, metabolic pathways linked to downregulated genes include those related to the addiction to cocaine, morphine or amphetamine, to PD, cAMP signaling and neuroactive ligand–receptor interaction. (Fig. S17). Pathways linked to downregulated genes following analysis of HD mouse brain hemisphere datasets are shown in Fig. S18.

Pathway analysis of genes upregulated in human NPCs datasets showed enrichment of the AGE-RAGE signaling pathway and regulation of inflammatory mediators of TRP channels, whereas that of downregulated genes showed enrichment of pathways involved in Hippo, Wnt, TGF-beta, MAPK and Rap1 signaling, axon guidance, cancer and glutamatergic synapse (Fig. S19). Pathway analysis of genes found downregulated in human transcriptomics data of peripheral blood showed upregulation of endocytosis, reduced lysosome, MAPK or RAP1 signaling, and reduced caffeine metabolism. (Fig. S20). Pathway analysis of upregulated genes included enrichment of the glutamate–glutamine pathway, protein export and ubiquitin-mediated proteolysis, protein processing in the endoplasmic reticulum and those related to AD (Fig. S20). Pathway analysis of genes downregulated in human VHCN included pathways having a role in AD, PD and HD, thermogenesis and oxidative phosphorylation, the GABAergic synapse pathway and others, whereas pathway analysis of genes upregulated include pathways involved in cancer, ECM receptor interaction, focal adhesion and axon guidance (Fig. S21). Details on the GSE IDs used for analysis of mouse and human gene expression GEO datasets are provided in Table S1.

### Proteomics analysis of tissue and serum samples from HD patients and model systems reveals pathways that are deregulated in disease

Proteomics HD mouse datasets analyzed include those of cortex, striatum, hippocampus (Skotte et al., 2018) and synaptosomes from striatum (Sapp et al., 2020) (see Table S1 for details). Proteomics HD patient datasets analyzed include those obtained from superior frontal gyrus (SFG) tissue (Ratovitski et al., 2016), middle frontal gyrus and visual cortex (Schönberger et al., 2013), the striatum and cortex (Sorolla et al., 2008), as well as from embryonic stem cells (ESCs) (McQuade et al., 2014). Pathway analysis of proteomics datasets of mouse cortex, hippocampus, striatum and synaptosome-striatum was carried out from published literature (details provided in Table S1). We found considerable overlaps between datasets of mouse cortex, hippocampus and striatum (Fig. 5E), with overlapping pathways including adrenergic signaling in cardiomyocytes, pathways having a role in alcoholism, aldosterone synthesis and secretion, amphetamine addiction, amyotrophic lateral sclerosis (ALS), apelin signaling pathway, arginine and proline metabolism, calcium signaling pathway, cholinergic synapse, circadian entrainment, cocaine addiction, dopaminergic synapse, fatty acid degradation, glutamatergic synapse, long-term potentiation, morphine addiction, oxytocin signaling pathway, proteasome, protein processing in the endoplasmic reticulum and cAMP signaling pathway (Fig. S22a). Human datasets analyzed include those of middle frontal gyrus and visual cortex, striatum and cortex, ESCs and SFG collected from the literature (details provided in Table S1). A commonality analysis for the pathways was also carried out (Fig. 5F). Pathways that were common to all datasets include those linked to fructose and mannose metabolism, African trypanosomiasis, antigen processing and presentation, apoptosis, fluid shear stress and atherosclerosis, influenza a, legionellosis, malaria, measles, phagosome, spliceosome, synaptic vesicle cycle, toxoplasmosis, vibrio cholerae infection, glycolysis/gluconeogenesis, TCA cycle, glutathione metabolism (Fig. S22b). Furthermore, we pooled pathways of the mouse datasets to compare them with those obtained from human datasets, finding considerable overlap (72 pathways) between mouse and human datasets (Fig. 5G). These pathways include metabolic pathways – such as those having a role in the metabolism of amino acids, fatty acids, butanoate, TCA cycle and others – and pathways having a role in AD, PD and non-alcoholic fatty liver disease were also found. However, endocrine and hormone, pathways of neurotransmitters in synapses and signaling pathways predominated the overlapping pathways.

### Commonality analysis of transcriptomics, proteomics and metabolomics datasets from HD patients and, yeast and mouse model systems shows pathways with potential disease implications

**Fig. 6.**
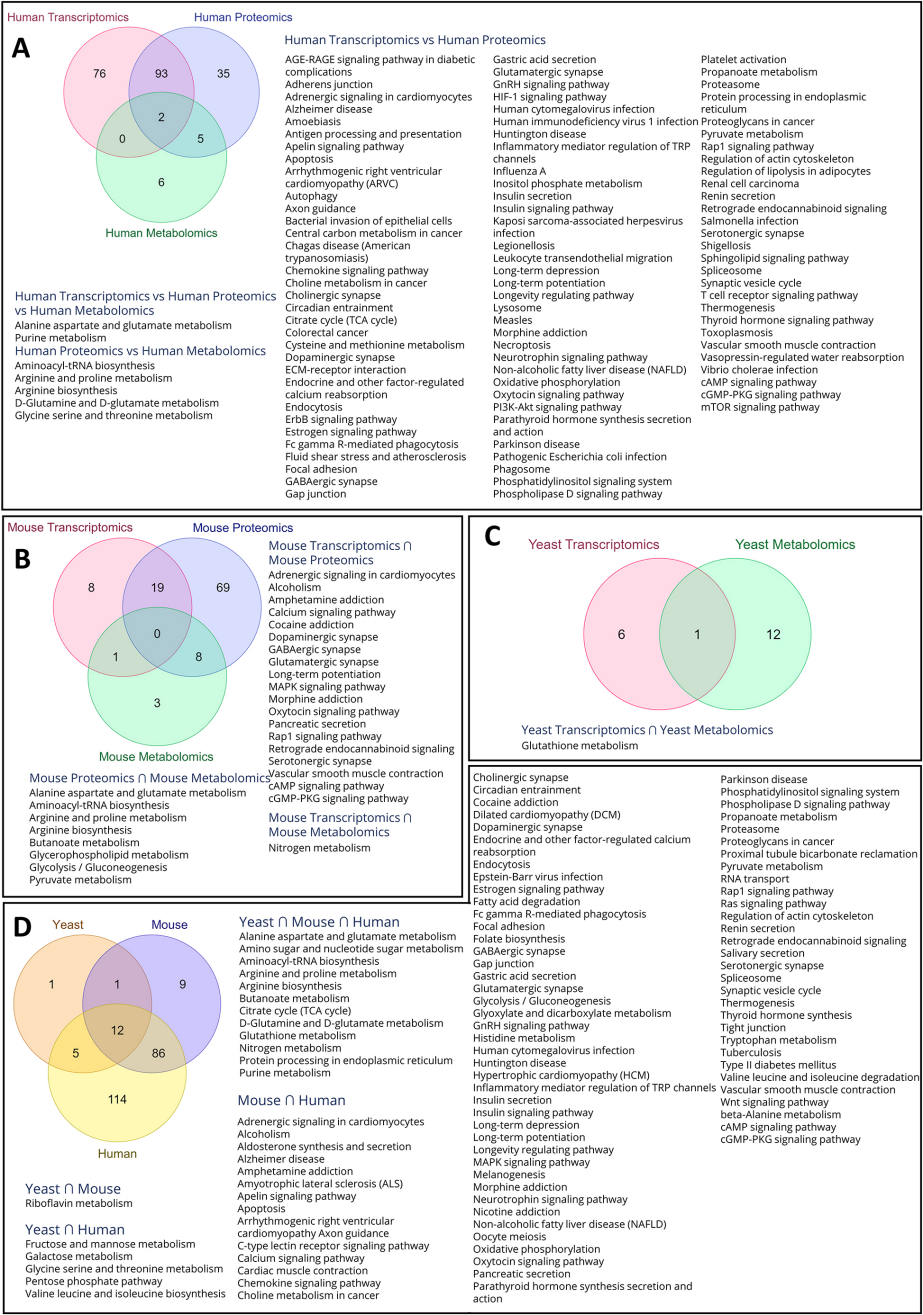
**Multi-omics and cross organism comparisons.** (A-C) Venn diagrams show the intersection of commonly deregulated pathways in the transcriptomics, proteomics and metabolomics datasets of HD patients (A), and mouse (B) and yeast (C) HD models. (D) Common pathways that are deregulated between HD model organisms (yeast and mice) and HD patients.

Commonality analysis of human datasets showed two shared pathways for all the three omics datasets, i.e. the alanine, aspartate and glutamate metabolism as well as the purine metabolism pathway (Fig. 6A). Proteomic and metabolomic datasets shared five pathways, (Fig. 6A), i.e. aminoacyl-tRNA biosynthesis, arginine and proline metabolism, arginine biosynthesis, glutamate–glutamine metabolism as well as glycine serine and threonine metabolism. Whereas we found no shared pathways between transcriptomics and metabolomics datasets, the human transcriptomics and proteomics datasets shared 93 pathways (Fig. 6A). These pathways involve synaptic transmission, pathways in neurodegenerative diseases, signaling pathways, inflammation and metabolic pathways.

Our analysis of the mouse datasets showed 19 shared pathways between transcriptomics and proteomics datasets, eight shared pathways between proteomics and metabolics datasets, but only one shared pathway between transcriptomics and metabolomics datasets (Fig. 6B). Commonality analysis of yeast transcriptomics and metabolomics datasets identified glutathione metabolism as the only overlapping pathway (Fig. 6C). With 12 shared pathways, the pooled multi-omics datasets of HD patients, and HD mouse and yeast models showed substantial overlap (Fig. 6D). These pathways include the alanine, aspartate and glutamate metabolism; amino-sugar and nucleotide-sugar metabolism; aminoacyl-tRNA biosynthesis; arginine biosynthesis; nitrogen, butanoate, glutathione, and purine metabolisms; D-glutamine and D-glutamate metabolism; TCA cycle; and protein processing in the endoplasmic reticulum. The HD patient and HD mouse model datasets share 86 pathways, including those regarding synaptic transmission, and those having a role in neurodegenerative diseases, signaling pathways, inflammation and metabolic pathways. HD patient and HD yeast model datasets shared five pathways, i.e. those linked to metabolisms of fructose and mannose; galactose; glycine, serine and threonine; the pentose phosphate pathway; valine, and leucine and isoleucine biosynthesis. The HD mouse and yeast model datasets shared one – the riboflavin – pathway (Fig. 6D). Taken together, our analysis found deregulated metabolic and signaling pathways that are shared between yeast, human and mouse, whereas inflammation, synaptic transmission as well as pathways that have a role in neurodegenerative diseases are deregulated in mice and human.

### Validation of certain metabolic pathways deregulated in HD patients and HD model systems indicates that they modulate HTT protein aggregation in the yeast model of HD

The HD yeast model 72Q was used for all further experiments. We identified shared metabolic pathways that are deregulated in HD patients as well as mouse and yeast models of HD, but also for those deregulated and shared between at least two of the systems. We found some shared pathways between HD patients and the mouse HD model, which include the metabolism of glutathione, tryptophan, glyoxylate and dicarboxylate, TCA cycle and longevity-regulating pathway (Fig. 6D). To evaluate the role of metabolites or gene knockout of specific genes from the deregulated pathways on aggregation of HTT protein, we used the yeast model 72Q. Our analysis showed 12 pathways that are shared between all datasets, and five and 86 pathways that are shared between two datasets, i.e. between yeast and human, and mouse and human datasets, respectively (Fig. 6D). However, some pathways were unique to the dataset of only one species. We then asked whether the pathways shared between human and mouse, or those unique to human or mouse are also conserved in yeast as – in that case – any effect of perturbation of such pathways, could be studied by using the yeast HD model. Our results (Fig. 6D) suggest that, despite the pathways not being identified in yeast as deregulated at transcriptomics or metabolomics level, the yeast model system can be used to investigate the role of these pathways regarding HTT protein aggregation. This might also be due to the depth of coverage during transcriptomics or relative abundance of metabolites during metabolomics analysis when a large number of metabolites are being targeted.

**Fig. 7.**
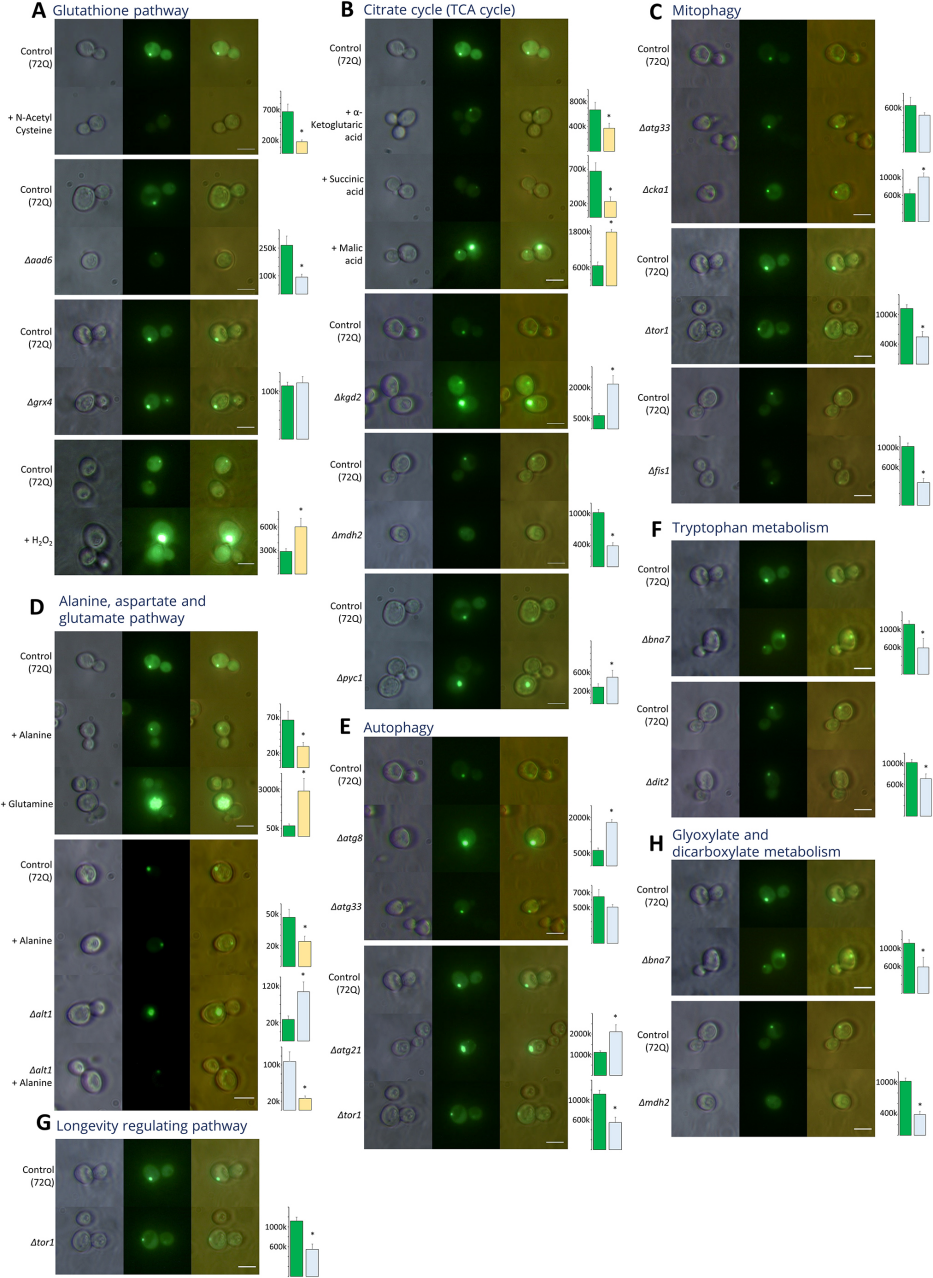
**Yeast fluorescence studies, highlighting the role of different metabolites and gene products in relation to deregulated metabolic pathways and HTT protein aggregation.** (A-H) The listed metabolic pathways were investigated following the indicated addition of certain metabolites or the knockout of certain genes. Glutathione pathway (A), TCA cycle (B), mitophagy (C), alanine aspartate and glutamate pathway (D), autophagy (E), tryptophan metabolism (F), longevity regulating pathway (G), glyoxylate and dicarboxylate metabolism (H). Scale bars: 10μm. Bar diagrams represent the corrected total HTT fluorescence (arbitrary units) comparing HTT fluorescence in control (green), knockout (Δ, blue) and treated (yellow) samples. *P<0.05.

As mentioned above, the TCA cycle is shared between the transcriptomics datasets of HD patients and HD yeast systems (Fig. 5D), as well as between transcriptome and proteomics datasets from HD patients (Fig. 6A). To understand the role of the TCA cycle in HD, we carried out metabolic addition as well as knockout experiments. In 72Q, addition of α-ketoglutaric acid or succinic acid led to a significant reduction of HTT protein aggregates (Fig. 7B), whereas gene knockout of α-ketoglutarate dehydrogenase (*KGD2*) substantially increased HTT protein aggregation (Fig. 7B). Further, gene knockout of pyruvate carboxylase 1 (*PYC1*) also significantly increased HTT protein aggregation (Fig. 7B). Gene knockout of cytoplasmic malate dehydrogenase 2 (MDH2), a cytosolic isoform of malate dehydrogenase abrogated HTT protein aggregates (Fig. 7B). Our data also showed that metabolites of the TCA cycle or enzymes in the pathway modulates protein aggregation of HD (Fig. 7B).

We found that human, yeast and mice metabolomics datasets show deregulation of alanine, aspartate and glutamate, as well as glutamate–glutamine pathways (Fig. 3G). The alanine, aspartate and glutamate pathway was shared between human transcriptomics, proteomics and metabolic datasets (Fig. 6A). Human and mouse proteomics data also showed alanine, aspartate and glutamate as a shared pathway (Fig. 5G). Hence, we tested the effect metabolic addition of glutamine and alanine as well as gene knockout of alanine transaminase (*ALT1*) has on HTT protein aggregation in the yeast model system. Addition of glutamine led to a significant increase in protein aggregates, whereas addition of alanine reduced protein aggregation (Fig. 7D). Further, knockout of *ALT1*, the cytosolic isoform of alanine transaminase, led to a significant increase in protein aggregation (Fig. 7D). Addition of alanine to *ALT1* knockout yeast led to a significant decrease in protein aggregation (Fig. 7D, *Δalt*+alanine). Therefore, our results show the importance of these pathways in modulating HTT protein aggregation.

Our analysis showed deregulation of glutathione metabolism in yeast transcriptomics (Fig. S16) and metabolomics datasets (Fig. 3B), as well as in mouse and human proteomics datasets (Fig. 5G). N-acetyl cysteine, which replenishes the intracellular glutathione pool, led to significant reduction in protein aggregation (Fig. 7A). To reinforce our observations of metabolic addition experiments, we carried out experiments using yeast gene knockouts for aryl-alcohol dehydrogenase (*AAD6*) and glutathione-dependent oxidoreductase and glutathione S-transferase (*GRX4*). Whereas knockout of *AAD6* reduced protein aggregates, that of *GRX4* did not cause any significant change in protein aggregation (Fig. 7A). Further, to evaluate the role of oxidative stress on HTT aggregation in the yeast model we used H_2_O_2_, addition of which led to a significant increase in HTT protein aggregation (Fig. 7A). Taken together, our results show a crucial role of the cellular redox and glutathione systems in modulating HTT protein aggregates.

We found the tryptophan pathway to be shared between mouse and human proteome datasets (Fig. 5F). The role of tryptophan metabolism in HD and protein aggregation has been demonstrated previously (Stoy et al., 2005). We, therefore, carried out knockout studies of genes relevant to tryptophan metabolism in yeast. Knockout of kynurenine formamidase (*BNA7*) significantly reduced HTT aggregation, whereas knockout of N-formyltyrosine oxidase (*DIT2*) led to significant increase in HTT aggregation (Fig. 7F). Our results emphasize the importance of the tryptophan pathway in modulating HTT protein aggregates and its implication for disease. We found the glyoxylate and dicarboxylate metabolic pathway to be enriched only in the human proteomics dataset. Knockout of the genes *MDH2* and *BNA7* in the pathway led to the attenuation of protein aggregates (Fig. 7H). Moreover, MDH2 and BNA7 are component enzymes of the TCA and tryptophan metabolic pathways, respectively.

We found the longevity-regulating pathway is shared between mouse and human proteomics datasets (Fig. 5G). PIK-related protein kinase and rapamycin target (TOR1) is a part of the longevity pathway, and its knockout led to complete abrogation of HTT protein aggregates (Fig. 7G). The autophagy pathway was observed in human transcriptomics and proteomics datasets, and knockout of genes encoding autophagy-related protein 8 and 21 (*ATG8* and *ATG21*, respectively) led to significantly increased HTT protein aggregation (Fig. 7E). TOR1 and ATG33 negatively regulate autophagy pathway (Noda and Ohsumi, 1998; Pattingre et al., 2008; Kanki and Klionsky, 2010). Knockout of *TOR1* led to attenuation of HTT protein aggregates, whereas knockout of *ATG33* did not cause any significant changes to aggregation of HTT (Fig. 7E). The mitophagy pathway was found to be enriched in the human transcriptomics PBMC dataset (Fig. S20). Previous studies have also shown an important role for mitophagy in HD (Fivenson et al., 2017). Our results show that knockout of mitochondrial fission 1 (*FIS1*), which is involved in mitochondrial fission, led to significantly decreased HTT protein aggregation (Fig. 7C). Knockout of casein kinase II subunit alpha (*CKA1*) also led to increased HTT protein aggregation (Fig. 7C).

Taken together, our results show an important role for metabolic pathways, such as the TCA cycle; glutamate and tryptophan metabolisms; and autophagy, mitophagy, glyoxylate, and dicarboxylate cycles in modulating protein aggregation. The yeast model concurs with the feasibility of testing the role of deregulated pathways in HD patients or other HD model systems, even if these are not deregulated in yeast.

## DISCUSSION

Mitochondrial dysfunction and metabolic rewiring are associated with HD (Quintanilla and Johnson, 2009). Studies have shown dysfunction of electron transport chain complexes I, II and III in the mitochondria of HD patients (Koroshetz et al., 1997). Metabolic pathways lead to the cumulative changes observed in genomics, epigenomics, transcriptomics and proteomics (Dettmer and Hammock, 2004) and, hence, might be interesting targets to treat several neurodegenerative diseases. Further, changes in metabolic profiles also lead to changes in transcriptomics and other responses within different cell types (Castegna and Menga, 2018). In HD patients, function of PPARGC1A (also known as PGC-1α) – one of the key regulators of the mitochondrial transcription factor A (TFAM) – is impaired (Johri et al., 2013). Levels of TFAM protein directly correlate with the copy number of mtDNA, and reduction in the copy number of mtDNA and/or nuclear DNA is directly associated with loss of mitochondrial function, leading to motor symptoms in HD patients (Petersen et al., 2014). Mitochondrial dysfunction leads to increased reliance to glycolysis, leading to a decreased ratio of NADH:NAD, which is ascribed to reduced activity of PDH (Ferreira et al., 2011; Smith et al., 2018). Consistent with these findings, our multi-omics analysis described here, as well as previous studies, show the deregulation of glycolysis, TCA cycle and pyruvate metabolism in HD datasets (Borovecki et al., 2005; Tauber et al., 2011; Durrenberger et al., 2012; Ratovitski et al., 2016; Schönberger et al., 2013; McQuade et al., 2014; Tsang et al., 2006). Previous studies also found that dichloroacetate, an inhibitor of PDH kinase (PDK), or lipoic acid, an activator of PDH, improve cognitive, behavioral and motor function in the mouse model of the disease (Holmquist et al., 2007; O'Hara et al., 2019). Similarly, metformin also improves memory, cognitive, behavioral and motor function in the HD mouse model and in HD patients (Ma et al., 2007; Rotermund et al., 2018). Elevated levels of lactate are associated with many neurodegenerative diseases, such as PD, HD, AD and ALS (Liguori et al., 2016; Martin et al., 2007; Siciliano et al., 2002; Yamamoto et al., 1997), and previous studies have shown that aggregation of amyloid beta peptides is increased in the presence of lactate at pH levels below 7 (acid) (Su and Chang, 2001; Xiang et al., 2010). These results reiterate that mitochondrial dysfunction and metabolic rewiring can lead to impaired energy metabolism and disastrous consequence in the process of HD.

In the yeast model of HD, metabolic addition of α-ketoglutarate and succinate significantly reduced, whereas knockout of *KGD2* significantly increased HTT protein aggregation. High activity of α-ketoglutarate dehydrogenase, an enzyme in the TCA cycle that links TCA to amino acid biosynthesis has been shown in a HD cybrid cell model (Ferreira et al., 2011). Studies have shown that the activity of succinate dehydrogenase (SDH) is very high in human and mouse cortexes (Naseri et al., 2015). However, HD patient lymphoblastoid cells showed increased activity of PDH, isocitrate dehydrogenase 1 (IDH1) and SDH, whereas that of MDH was found to be significantly reduced compared to those from healthy controls (Naseri et al., 2016). Our results show that knockout of the pyruvate carboxylase (*PYC1*) gene lead to a significant increase in HTT protein aggregation. This might be caused by reduced flux through the TCA cycle, as the concentration of oxaloacetate might be limiting under these conditions. Further, the addition of malate significantly increased under this condition, whereas knockout of the cytosolic malate dehydrogenase gene *MDH2* significantly reduced aggregation of HTT protein (Fig. 7B). Studies have also shown that increased levels of malate are associated with neurodegeneration (Patassini et al., 2016).

Our analysis showed deregulation of alanine, aspartate and glutamate metabolism in almost all the human, mouse and yeast multi-omics datasets. Moreover, levels of alanine were low in HD patients and HD mouse model systems. In the HD yeast model, addition of alanine led to decreased aggregation of HTT protein, whereas knockout of *ALT1* significantly increased it. HTT protein aggregation in response to knockout of *ALT1* could be mitigated by the addition of alanine (Fig. 7D). Previous studies have shown decreased levels of alanine and isoleucine in HD patients, whereas alanine and leucine levels are elevated in pre-symptomatic cases (Reilmann et al., 1995; Underwood et al., 2006). Activity of mitochondrial and cytosolic alanine aminotransferase was found to be significantly increased in the putamen of HD patients (Carter, 1984). A previous study of metabolic biomarker analysis of AD, PD and ALS metabolomics data, found L-glutamine, alanine, choline and N-acetyl-aspartate to be shared between all three diseases (Kori et al., 2016). Deregulation of alanine, aspartate and glutamate metabolisms is attributed to decreased glucose levels, which is concomitant with the energy deficits in the brain and peripheral organs of HD patients (Kori et al., 2016). HD patient cybrid cells showed elevated levels of mitochondrial glutamate and reduced levels of alanine but no changes in glutamine and aspartate levels (Ferreira et al., 2011). Taken together, alanine might be an important metabolite to modulate HTT protein aggregation in HD.

The glutamate–glutamine metabolism was found to be deregulated in metabolomics datasets of human, mouse and yeast as well as in human proteomics datasets. In mammalian brain, astroglia take up glutamate and secrete glutamine (Höltje et al., 2008). Glutamate transporters are downregulated in HD, resulting in reduced glutamate uptake followed by glutamate-mediated excitotoxicity (Behrens, 2002; Raymond, 2003). Transgenic HD mouse models show changes in the levels of glutamate transporters, glutamine synthase and extracellular glutamine (Behrens, 2002; Skotte et al., 2018). Indeed, glutamatergic synapses and signaling are deregulated, as shown by analysis of transcriptomics and proteomics datasets of HD mice and HD patients (Fig. 5D,G). Our analysis showed that levels of glutamine are elevated in mouse and yeast metabolomics datasets but decreased in those from HD patients; and addition of glutamine in yeast model of HD led to significantly increased aggregation of HTT protein (Fig. 4A,B). Elevated levels of glutamine are indicative of an increase in energy-rich metabolites (Cruzat et al., 2018), and previous studies have shown that they can activate mTOR1 in mammalian cells (Meng et al., 2020). Increased TOR1 activity is associated with reduced autophagy (Neufeld, 2010). Consistent with this, knockout of TOR1 attenuated HTT aggregates in our study. Increased availability of glutamine could, therefore, aid the efficient translation of polyQ HTT protein, resulting in increased aggregation. Similarly, transglutaminase was shown to modulate protein aggregation, and knockout of transglutaminase reduces protein aggregate load (Karpuj et al., 2002; Mastroberardino et al., 2002).

Our study here showed deregulation of the glutathione pathway in yeast, human and mouse datasets. Consistent with this, we observed deregulation of the pentose phosphate pathway (PPP) – the conversion of glucose to ribose-5-phosphate – in the human proteomics dataset. Our results show that HTT protein aggregation in yeast was significantly increased following addition of H_2_O_2_, whereas it was significantly reduced after addition of N-acetyl cysteine. Mitochondrial dysfunction, leading to elevated levels of ROS and the concomitant change of the redox status, has been reported in HD (Zheng et al., 2018), and HD cybrids show decreased activity of glucose-6-phosphate dehydrogenase, a key and rate-limiting enzyme in the PPP (Ferreira et al., 2011). Reduced activity of the PPP correlates with reduced cellular NADPH levels, both essential for maintaining the intracellular glutathione pool (Dringen et al., 2007). An increased ratio between extracellular cysteine and cystine was found to replenish mitochondrial NADH, leading to better ability of neurons isolated from AD mice to confront oxidative stress (Dong et al., 2019). Another study showed that addition of flavin mononucleotide (FMN) to an AD yeast model increases survival during chronological aging and improves tolerance of oxidative stress, with the mechanistic action of FMN attributed to increased levels of cellular NADPH (Chen et al., 2020). A genome-wide suppressor study, searching for genes that ameliorate toxicity of mutant HTT in yeast, identified glutathione peroxidases to catalyze reduction of H_2_O_2_ and lipid peroxides (Mason et al., 2013). Furthermore, genetic and pharmacological approaches reiterated the efficacy of glutathione peroxidase in mitigating HD in mammalian cell and fly models of HD (Mason et al., 2013). Taken together, our results, together with previous reports, show that antioxidants, such as N-acetyl cysteine, as a dietary supplement might improve management of HD (Wright et al., 2015).

Multiple datasets obtained from HD patients and HD mouse models show deregulation of tryptophan metabolism (Skotte et al., 2018; Herman et al., 2019; Graham et al., 2016; 2018) (Fig. S14a, S22a). In neurodegenerative diseases, such as HD, PD, AD or glaucoma, microglia are activated and upregulate tryptophan metabolism in brains of human patients, mouse and rat models. (Fiedorowicz et al., 2019; Guillemin and Brew, 2002; Yang et al., 2017; Zinger et al., 2011). Products of tryptophan metabolism involve neurotoxic metabolites that activate NMDA receptors, thereby inducing neuronal cell death through excitotoxicity (Jhamandas et al., 2000). Our results show that knockout of *BNA7* significantly reduced HTT protein aggregation, whereas knockout of *DTI2* significantly increased it. Previous studies have also shown a role of tryptophan metabolism in HTT protein aggregation (Guillemin et al., 2003). Gene knockout of *Ido1*, affecting the tryptophan pathway, was shown to be protective in a mouse model of HD (Mazarei et al., 2013), and our results largely support previously published results.

We also found that mitophagy is impaired in HD, and impaired mitophagy reduces mitochondrial quality resulting in increased ROS production (Franco-Iborra et al., 2021; Zheng et al., 2018). We have shown here that knockout of *CKA1* significantly increased protein aggregation, which may be attributed to impaired mitophagy, and previous studies found CKA2 to be essential for autophagy (Kanki et al., 2013). HTT aggregates interact with mitophagy machinery and impair mitophagy (Franco-Iborra et al., 2021). By using a yeast model of HD, we were able to show that knockout of *FIS1* leads to significant reduction of HTT aggregates. FIS1 is involved in mitochondrial fission that impairs mitochondrial function (Reddy, 2014). During inflammatory responses, mitochondrial fission results in compromised mitochondrial function and upregulation of glycolysis (Guido et al., 2012). Hence, inhibition of mitochondrial fission might have beneficial therapeutic effects in HD. Consistent with compromised mitochondrial function, we found glycolysis to be upregulated in multiple datasets obtained from HD patients, mouse and yeast HD models, which reiterates the importance of mitochondrial health and function in HD.

HTT aggregates are cleared mainly through the autophagy pathway (Yamamoto et al., 2006). In addition, previous studies have shown a role for HTT protein in selective autophagy in *Drosophila* and mouse CNS (Ochaba et al., 2014; Ehrnhoefer et al., 2018). Consistent with this, accumulation of mutant HTT is associated with defects in basal autophagy as well as with cargo loading during selective autophagy (Ehrnhoefer et al., 2018). Indeed, the analysis of human transcriptomics and proteomics datasets showed deregulation of autophagy (Borovecki et al., 2005; Ratovitski et al., 2016) (Fig. 5G, Fig. S20). Our *ATG8* and *ATG21* knockout studies showed increased protein aggregations (Fig. 7E), and previous studies in yeast also have shown a role for autophagy in the clearance of aggregated protein (Hofer et al., 2018). In addition, our current study found that inhibition or knockout of *TOR1* leads to attenuated protein aggregation. TOR1 has also been shown to negatively regulate autophagy and mitophagy (Liu and Okamoto, 2018), and inhibition of mTOR might have therapeutic potential in the treatment of AD, PD, ALS and HD (Ravikumar et al., 2004; Van Skike et al., 2018; Zhu et al., 2019). The autophagy pathway has also been related to longevity (Bareja et al., 2019) and previous studies have demonstrated that fasting or calorie restriction upregulates autophagy leading to an increased life span (Chung and Chung, 2019). Our study here shows that impaired autophagy might have a role in HD progression.

Overall, we found deregulation of signaling, metabolic and other pathways in HD. Many deregulated pathways overlapped between HD patients, mouse and yeast models of the disease, whereas some of the pathways were unique to patients or model systems. Metabolic addition and gene-knockout experiments targeting selected pathways showed their role in modulating HTT protein aggregation in the yeast model of HD. Even regarding deregulated pathways unique to HD patient or HD model mouse datasets, it was possible to employ the HD yeast model to understand their role in HTT aggregation – provided the pathway was conserved in yeast. Taken together, our results show a crucial role for the alanine, aspartate and glutamate; glutamine and glutamate; glutathione; longevity; mitophagy and autophagy pathways in modulating protein aggregation in HD model yeast, with potential implications for HD. In addition, pathways regulating glycolysis or reduced mitochondrial function, the glutamate–glutamine pathway, and the nucleotide pathway are associated with inflammation and immune metabolism. Targeting these pathways might help to mitigate inflammation and to improve prognosis. Further, targeting some of these signaling and metabolic pathways might help to activate autophagy, thereby leading to clearance of HTT aggregates and improvement of prognosis. Overall, our results – concomitant with our previously published findings regarding atrophy and volume changes in the brain of pre-symptomatic HD patients (Thota et al., 2021) – show that, by modulating deregulated pathways early in genetically susceptible individuals, might improve management of the disease.

### Conclusions

Our metabolomics analysis of HD patients and yeast models of HD showed a number of deregulated metabolic pathways. Considerable overlap in deregulated pathways was observed between HD yeast models expressing different polyQ lengths. Comparative analysis of data obtained from our HD patient cohort and yeast datasets compared with those from the literature showed considerable overlap between the different datasets.

Further, transcriptomics analysis of GEO datasets obtained from HD patients, mouse and yeast showed deregulated pathways shared between them. Mouse and human proteomics datasets also showed many shared pathways. HD yeast and mouse models, and HD patient metabolic datasets also showed many shared deregulated pathways. Commonality analysis of pooled pathways determined from multi-omics datasets of HD patient, mouse and yeast HD models also showed overlapping pathways – some were observed between at least two multi-omics data sets, whereas some were unique to only one.

By using a yeast model of HD, we also investigated the role overlapping pathways have on protein aggregation, and probed whether pathways found to be deregulated in two of the systems or unique to a single system can be validated by using yeast models. The TCA cycle metabolites α-ketoglutarate and succinate decreased protein aggregation, whereas knockout of *KGD2* increased it. Analysis by using yeast models shows that metabolic addition of alanine decreased HTT aggregation, whereas knockout of *ALT1* increased it. Addition of glutamine significantly increased aggregation of HTT as did knockout of genes relevant in the tryptophan metabolism. HTT aggregation increased in response to H_2_O_2_-induced oxidative stress, whereas a significant decrease was found in response to the ROS scavenger N-acetyl cysteine. Moreover, whereas knockout of genes playing a role in autophagy or mitophagy pathways increased HTT aggregation, knockout of *FIS1* decreased it. Our results show that metabolites and proteins relevant in several deregulated pathways have a crucial role in modulating HTT aggregation and, therefore, implications regarding progression of HD. Even if a pathway deregulated in HD mouse or HD patients is not deregulated in the yeast HD model, yeast is still a good model system to validate the role of this pathway in HTT aggregation – provided the pathway is conserved. Taken together, our results and previous findings of volume change and atrophy in brains of pre-symptomatic HD patients (Thota et al., 2021; Kassubek et al., 2004; Squitieri et al., 2009), show that – in genetically susceptible individuals – modulation of deregulated pathways at the pre-symptomatic state might help to improve management of HD.

## MATERIALS AND METHODS

### Patients

For this study, 31 individuals were recruited from the Department of Neurology, Sri Sathya Sai Institute of Higher Medical Sciences (SSSIHMS), Bangalore, India. The cohort included eleven symptomatic HD patients, five pre-symptomatic HD patients, 11 individuals as familial controls and four random individuals as age- and sex-matched controls. The study was approved (approval number: SSSIHL/IEC/PSN/BS/2017/01) by the Joint Institutional Ethics committee (IEC) of Sri Sathya Sai Institute of Higher Learning (SSSIHL) and SSSIHMS, according to the ethical standards set out in the 1964 Declaration of Helsinki and its later amendments.

### Metabolomics

#### Patient serum samples: sample preparation and analysis

Serum samples from HD patients, familial controls, and age- and sex-matched controls were collected for metabolomics analysis; 50 μl was used for extraction. The procedure for metabolite extraction and analysis was adapted from our previous studies (for further information, see Bhagavatham et al., 2021; Pulukool et al., 2021).

Labelled L-tryptophan (Cambridge Isotope Laboratories, Inc, NLM-800) was used for normalization; Student's *t*-tests was used to assess data obtained from groups of control vs disease, control vs pre-symptomatic, control vs symptomatic and pre-symptomatic vs symptomatic groups, with the false discovery rate set as *P*≤0.25. Heatmaps for the significantly different levels of metabolites are shown in Fig. 1A-D. Significant metabolites were taken for metabolite set enrichment analysis (MSEA). Links between significant pathways found to be enriched and significantly different levels of metabolites are shown as a chord diagram (Fig. 1E). Details on detected metabolites available in Table S3.

### Yeast

#### Culture and transformation

Yeast strains BY4741 and BY4742 were used for the study. Both strains were revived and plated in 2% YPD yeast extract powder (#RM027); Peptone (#RM001); D-(+)-Glucose anhydrous (#GRM016); 2% Agar plate (#RM026) (all by Himedia). Cells from the revived colonies were each cultured in 50 ml YPD and grown at 30°C until OD_600_=0.8. Cells were then harvested and washed twice with double-distilled water (ddH_2_O) to remove traces of medium. Collected cell pellets were resuspended in 25 ml of electroporation buffer [0.1 M lithium acetate (#GRM1507), 10 mM Dithiothreitol (DTT) (#MB070) 10 mM Tris-HCl (#MB030), 1 mM EDTA (#RM1370, ddH2O) (all by Himedia), and left to incubate in a shaking incubator at 30°C, 200 rpm for 1 h. Cells were then spun down (for 5 min at 1500 ***g***), washed and resuspended in 200 μl 1 M sorbitol. A total of 8×10^8^ cells was taken for transformation with p426 25Q GPD, p426 46Q GPD, p426 72Q GPD and p426 103Q GPD (Addgene plasmids #1181, #1182, #1183 and #1184, respectively). 8×10^8^ cells were incubated on ice with 2 μg/ml of plasmid DNA for 5 min and transferred into a 0.2 cm electroporation cuvette (Bio-Rad, #1652086). The BIO-RAD Gene Pulser Xcell was used for transformation, set at 1.5 kV/25 μF/200 Ω. URA selection plates –comprising complete supplement mixture w/o URA (#G112); yeast nitrogen base w/o amino acids (#M878), D-(+)-glucose (#MB037) and 2% agar (#RM026) (all by Himedia) – were used to grow and select transformed colonies. This process was carried out for all four polyQ plasmids.

#### Yeast metabolite supplementation and knockout studies

All experiments involving metabolite supplementation were done using HTT 72Q-transformed BY4742 yeast strains. HTT 72Q-transformed cells were grown in URA selection medium for 6 h, following which 200 μl of the culture was transferred to fresh medium containing metabolites. Cells were then let to grow for 12 h at 30°C. The metabolites used for the study are α-ketoglutaric acid (5 mM; #75890), L-alanine (5 mM; #A7627), L-glutamine (5 mM; #G3126), L-(−)-malic acid (5 mM; #112577), N-acetyl-L-cysteine (5 mM; #A7250) and succinic acid (5 mM; #398055) (all by Sigma), added at the time of inoculation. At 1 h before imaging, a solution of 30% H_2_O_2_ (#107209, Merck) was added to the culture medium (final concentration of 0.3 mM H_2_O_2_).

Knockout studies were performed by using the HTT 72Q transformed BY4742 strain as a control. Yeast knockout strains were procured from the Dharmacon yeast knockout library (Product No. YSC1054) for genes *AAD6*, *ALT1*, *ATG8*, *ATG21*, *ATG33*, *BNA7*, *CKA1*, *DTI2*, *FIS1*, *GRX4*, *KGD2*, *MDH2*, *PYC1* and *TOR1*, and revived in 2% YPD with Geneticin (G418) sulfate (#sc-29065A, Santa Cruz Biotechnology). Knockout strains were then transformed with HTT 72Q as previously mentioned, and selected using a double selection, using with URA selection plates and Geneticin (G418) sulfate. Transformed colonies were selected and grown at the same time as control cells for 12 h.

After the above-described two procedures fluorescence imaging against GFP was carried out by using a Laben BM-3000 FLT microscope (excitation wavelength of 470 nm and an emission wavelength of 509 nm). Fluorescence images were quantified by analyzing the corrected total cell fluorescence (CTCF) by using ImageJ (*n*≥10); Student's *t*-test was performed for significance. Results are shown in Fig. 7A-H. For each experiment three biological replicates (*n*=3) were carried out.

### Filter retardation assay

The BY4742 yeast strain transformed with HTT-polyQ GFP plasmids (25Q, 46Q, 72Q or 103Q) was cultured overnight until OD_600_=0.8 (Fig. S7a). Cells at OD_600_=2 were lysed with acid-washed 0.5 μm glass beads and the lysate was clarified to remove cell debris. The filter retardation assay protocol for measuring aggregation phenotype of the HTT-polyQ proteins was adapted as previously described by Cohen et al. (2012), using the Bio-Dot microfiltration apparatus (Bio-Rad). Briefly, after cell lysis, whole-cell lysates were diluted 5× with Tris-buffered saline (TBS, 50 mM Tris-HCl pH 8.0 (#MB030), 250 mM NaCl (#MB023) (all by Himedia); protein concentrations were determined using the Bradford assay. The bio-dot apparatus was assembled with pre-soaked nitrocellulose membrane (0.2 µm, #10600016, GE Health care) in 2% (w/v) SDS-containing TBS buffer. Aliquots of polyQ protein (50 µg) in TBS buffer containing 2% (w/v) SDS were heated to 100°C for 10 min and loaded in triplicates; vacuum was applied until filtering of the protein samples. Dot blot assays of polyQ GFP proteins were carried out using mouse anti-GFP antibody (B-2, #sc-9996, Santa Cruz Biotechnology; dilution 1:10,000) as primary antibody and goat anti-mouse IgG-HRP antibody (GeNie #105502C0619; dilution 1:5000) as secondary antibody. Visualization was done using WesternBright enhanced chemiluminescence (ECL) HRP substrate (K-12045-D20, Advansta, USA) in Syngene Gbox F3. The experiment was performed in three biological replicates (*n*=3).

### Spot assays

Viability of yeast was measured using spot analysis. The log-phase (OD_600_ ∼0.8) yeast culture was normalized to yeast at OD_600_ ∼0.5, 1:10 serial dilutions of were made using sterile water and 10 µl of each dilution was spotted on agar plates (Fig. S7c) in three biological replicates (*n*=3).

### Sample preparation and analysis of yeast

Samples of 8×10^6^ yeast cells each, transformed with 25Q, 46Q, 72Q or 103Q, were aliquoted, snap-frozen in liquid nitrogen and stored in −80°C for further use. Aliquots were used for the extraction of metabolites. We employed the freeze-thaw method for cell lysis. After three cycles of freeze and thaw, pellets were resuspended in extraction buffer (4:1 methanol: water); 2.5 μM of labelled internal standard [L-tryptophan 15N2, 98%; #NLM-800-PK), sodium pyruvate 13C3, 99% (#CLM-2440-PK) and D-Glucose, U-13C6, 99% (CLM-1396-PK) (all from Cambridge Isotope Laboratories, Inc., USA)] and unlabeled (+/−)-jasmonic acid (Sigma, #14631) were added to the extraction buffer (100 µl for 10 ml of extraction buffer). Each sample was then sonicated on ice for 30 s at a 5-10 pulse rate, following addition of 450 μl of cold chloroform and vortexed for 10 min. Thereafter, cold Millipore type1 water (150 μl per sample) was added and samples were vortexed for 10 min. Samples were then incubated at −20°C for 20 min, followed by centrifugation (1500 ***g***) for 10 min at 4°C, upon which a two-layer separation was seen. Both the layers were then transferred together into a new tube and left to dry in a speed vac (miVac) for ∼40 min. After drying the pellets were resuspended in methanol:water (50:50), vortexed and sonicated on ice for 5 min. Samples were then transferred to a conditioned [methanol:water (50:50)] Amicon filter column (Amicon Ultra 0.5 ml filter unit, #UFC500396, Merck Millipore, MA) and centrifuged for 6 h at 16,200 ***g*** at 4°C. Filtrates were collected into collection tubes, and each entire volume was used for quantitative metabolomics.

Of extracted metabolite samples, 2 μl were taken as the injection volume for quantitative metabolomics. Analyses were carried out using the Agilent 6490 iFunnel triple quadrupole LC–MS system. Samples analyses were carried out under positive and negative electron spray ionization, using an XBridge BEH Amide Column (130Å, 3.5 µm, 4.6 mm×100 mm; #186004868, Waters, Milford, USA). Details of detected metabolites are shown in Tables S4 and S5; LC–MS system run parameter details are available in Table S6.

Agilent Masshunter Quantitative analysis software (Version No. B.07.00) was used to obtain the abundance of each metabolite and to set the peak integration time with respect to the corresponding retention time for each metabolite. Acquired metabolites were normalized, using internal standard (+/−)-jasmonic acid for those acquired in negative mode and L-tryptophan for those acquired in positive mode.

To identify different levels of metabolite in control and sample, two-way Student's *t*-test analysis was carried out using MetaboAnalyst 5.0 (https://www.metaboanalyst.ca). Heatmaps for the different metabolites are shown in Fig. 2A-F. A metabolite was considered to be significant at a false discovery rate (FDR) value of ≤0.25. Significant metabolites for 25Q vs 46Q, 25Q vs 72Q and 25Q vs 103Q are compared in a Venn diagram (Fig. 3A). MSEA was carried out for these significant metabolites and significant metabolic pathways between these groups are compared in a Venn diagram (Fig. 3B). For details on detected metabolites, see Tables S4 and S5; for LC–MS system parameters, see Table S6.

### Metabolomics analysis

Previously published metabolome datasets were mined (sample details shown in Table S1) and used to analyze the metabolic profile for samples from HD patients. We used MetaboAnalyst 5.0 (https://www.metaboanalyst.ca), an online tool for MSEA. A hypergeometric test was employed as an enrichment method for the study. We used the Kyoto Encyclopedia of Genes and Genomes (KEGG) pathway database as reference for our enrichment analysis. Significant metabolic pathways were selected when FDR values were ≤0.25.

Pathways identified in yeast and human metabolomics datasets were pooled with pathways found to be enriched in this study, i.e. 25Q vs 46Q, 25Q vs 72Q and 25Q vs 103Q for yeast and control vs disease, control vs pre-symptomatic and control vs symptomatic for HD patients (Fig. 3C,F). Pooled pathways for human (Fig. 3F), and yeast (Fig. 3C) and mouse (Fig. 3D) were then taken for comparative analysis by using the online tool- Molbiotools multiple list comparator (https://molbiotools.com/listcompare.php) (as shown in Fig. 3G). Pathways shared between three [i.e. human, and mouse and yeast (Fig. 4A)] or two species [i.e. yeast and human (Fig. 4B)] were further constructed into networks by using Cytoscape (v3.8.2) (https://cytoscape.org/), together with their respective levels of significantly different metabolites.

### Transcriptomics data

Differential gene expression (DGE) analysis was done by using microarray datasets deposited at the National Centre for Biotechnology Information (NCBI) Gene Expression Omnibus (GEO) database. We retrieved the normalized series matrix file for each of the GSE IDs selected (sample details are shown in Table S1). The series matrix files were grouped into control and disease, with gene annotations and respective intensity values. The DGE analysis was done using NetworkAnalyst (https://www.networkanalyst.ca/), the online tool for comprehensive gene expression profiling and network visual analytics, which uses the limma package for analysis of gene expression datasets. Expression levels of genes were considered significantly different at a log2-fold change of 1 and *P*≤0.05. The implications significantly different levels of gene expression have on the disease were explored using the enrichment analysis tool Enrichr (https://maayanlab.cloud/Enrichr/), and the KEGG pathway database as a reference. Overlapping pathways, resulting from pathway enrichment were then selected based on their significance (*P*≤0.05). Significant pathways from the datasets were taken for comparative analysis using the online tool Molbiotools multiple list comparator (https://molbiotools.com/listcompare.php) (see Fig. 5A-D).

### Proteomics data

The proteomics profile datasets for HD in human and mouse were mined from the literature (for sample details see Table S1). The expression profile of the proteomics datasets was screened using the same selection criteria, i.e. log2 fold change and adjusted *P*≤0.05. Significant proteins were tested on having any implications on biological pathways by using the enrichment analysis tool Enrichr. Results of the enrichment analysis were then screened for significant pathways (adjusted *P*≤0.05). Significant pathways enriched in these datasets were taken for comparative pathway analysis of datasets, visualized in Venn diagrams (https://molbiotools.com/listcompare.php) as shown in Fig. 5E-G.

### Multi-omics comparative analysis

Significantly deregulated pathways identified after multi-omics analysis of human, mouse and yeast datasets were analyzed each for shared pathways (Fig. 6A-C), and pooled and analyzed for deregulated pathways shared in different species (Fig. 6D).

## Supplementary Material

10.1242/dmm.049492_sup1 Supplementary information
