# Supplementary material for: Integrated multi-omics analysis of Huntington disease identifies pathways that modulate protein aggregation
Source: Dis Model Mech. 2022 Oct 31;15(10):dmm049492. doi: 10.1242/dmm.049492 (PMC10655815; doi:10.1242/dmm.049492)
Supplement: Supplementary information [file dmm-15-049492-s1.pdf]

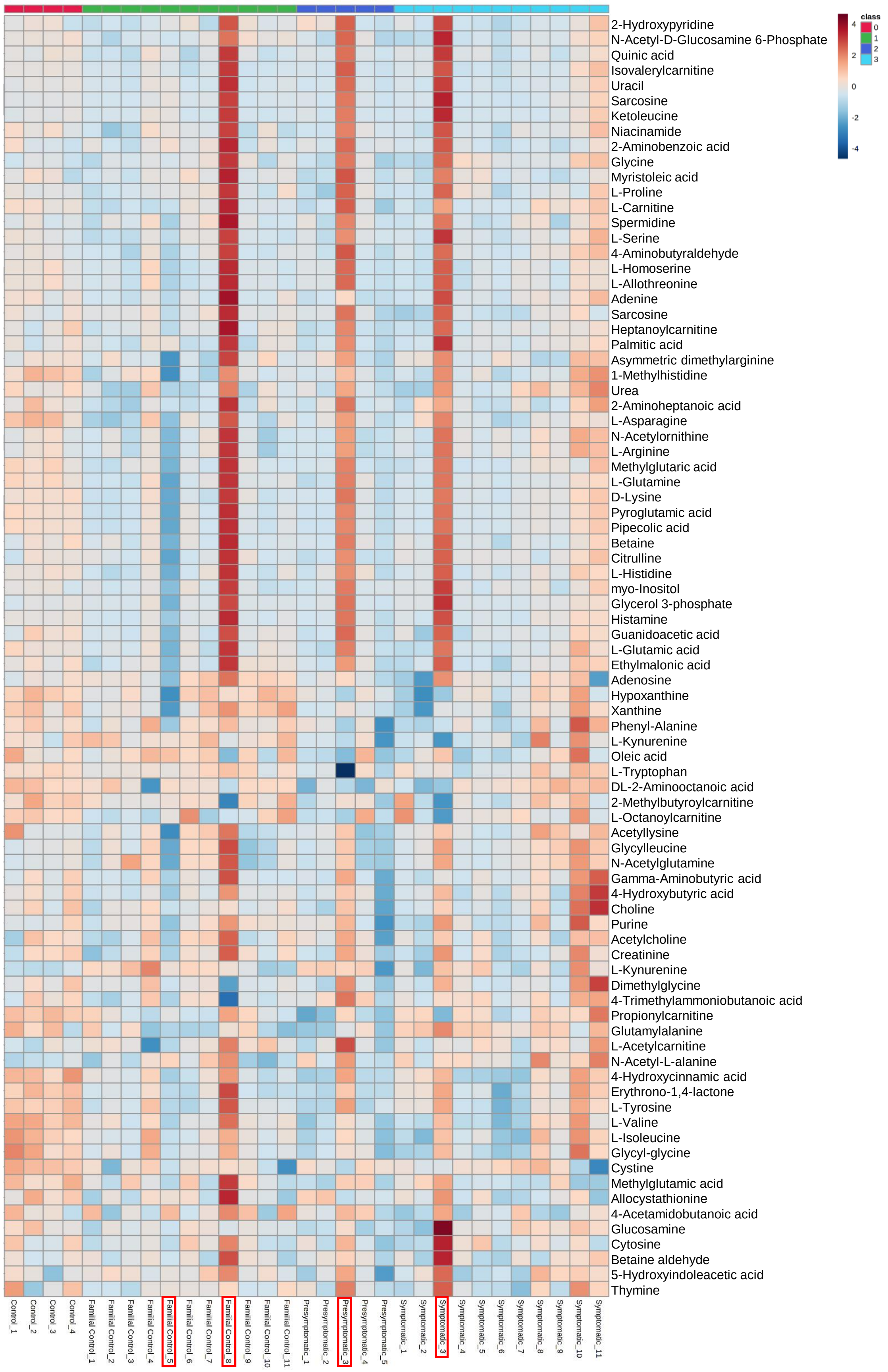

Fig. S1. Metabolomics- Heatmap

Heatmap showing metabolites detected in HD serum samples. The samples have been categorised into groups of control, familial control, presymptomatic and symptomatic. The outliers have been highlighted with red. Figures generated using MetaboAnalyst 5.0- https://www.metaboanalyst.ca/.

Disease Models & Mechanisms • Supplementary information

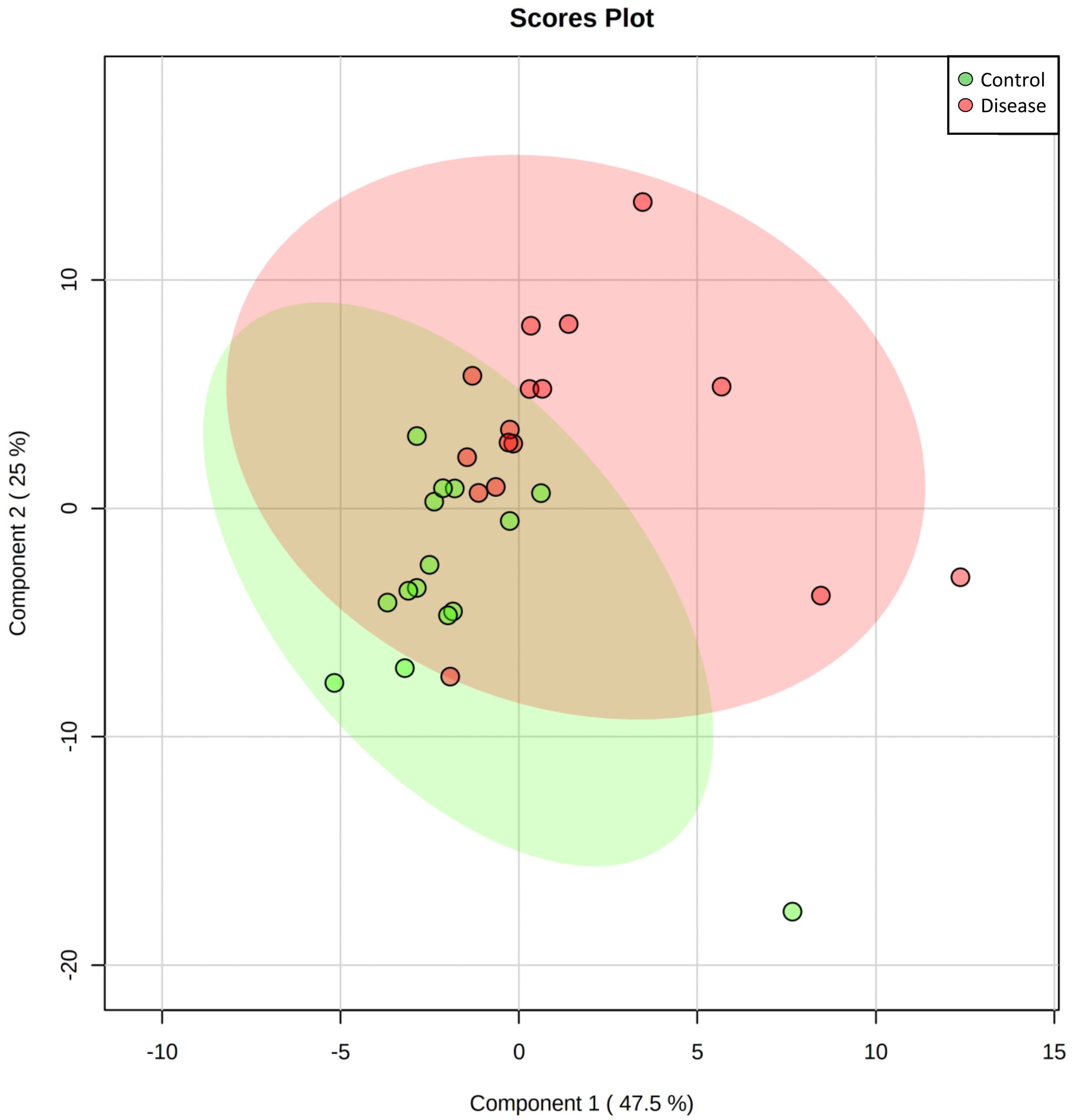

Fig. S2. Partial Least Squares Discriminant Analysis (PLS-DA)
PLSDA clustering show 2D score plot for metabolomic sample of HD serum of patient samples vs control with outliers. Figures generated using MetaboAnalyst 5.0- https://www.metaboanalyst.ca/.

Disease Models & Mechanisms • Supplementary information

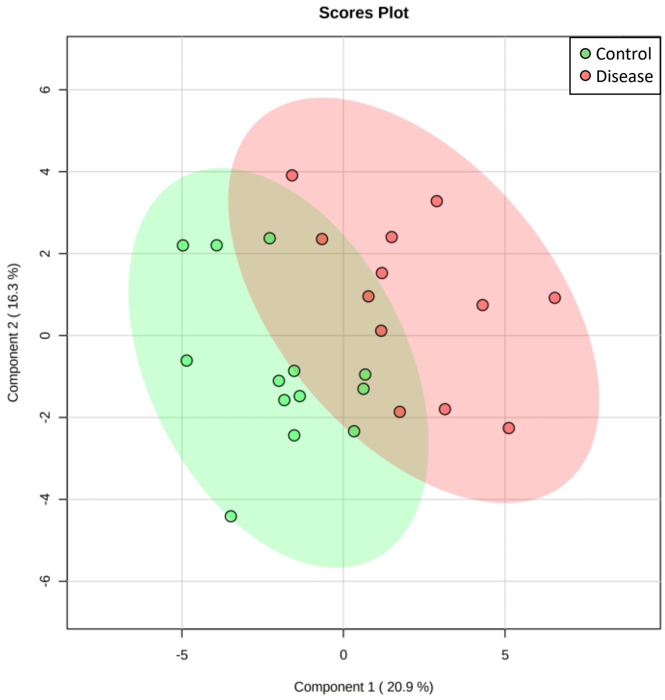

Fig. S3. Partial Least Squares Discriminant Analysis (PLS-DA)
PLSDA clustering show 2D score plot for metabolomic sample of HD serum of control vs disease group without outliers. Figure generated using MetaboAnalyst 5.0- https://www.metaboanalyst.ca/.

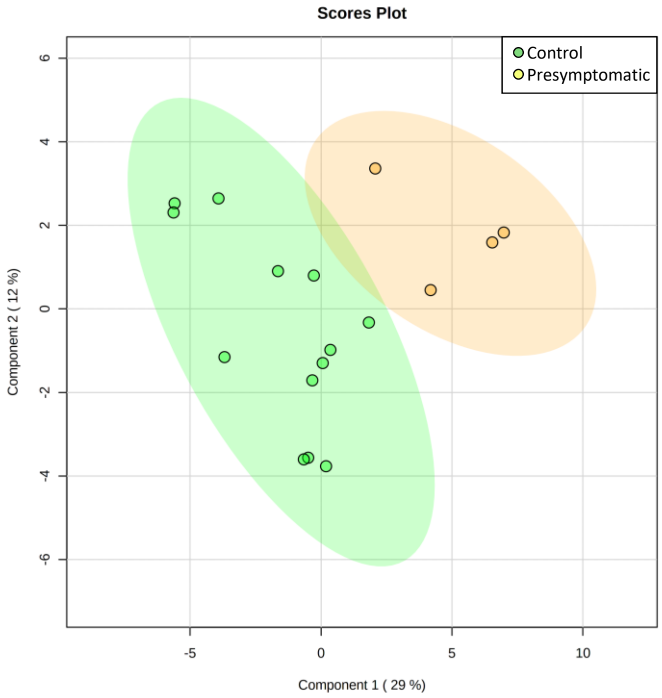

Fig. S4. Partial Least Squares Discriminant Analysis (PLS-DA)
PLSDA clustering show 2D score plot for metabolomic sample of HD serum of control vs presymptomatic. Figure generated using MetaboAnalyst 5.0- https://www.metaboanalyst.ca/.

Disease Models & Mechanisms • Supplementary information

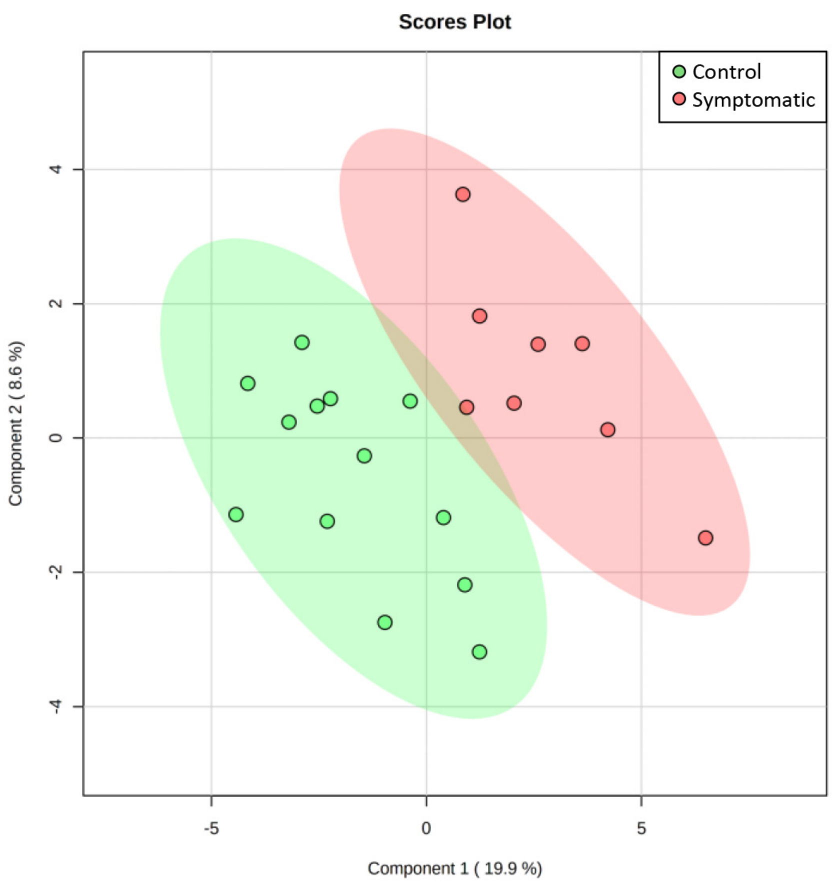

Fig. S5. Partial Least Squares Discriminant Analysis (PLS-DA)
PLSDA clustering show 2D score plot for metabolomic sample of HD serum of presymptomatic vs symptomatic. Figure generated using MetaboAnalyst 5.0- https://www.metaboanalyst.ca/.

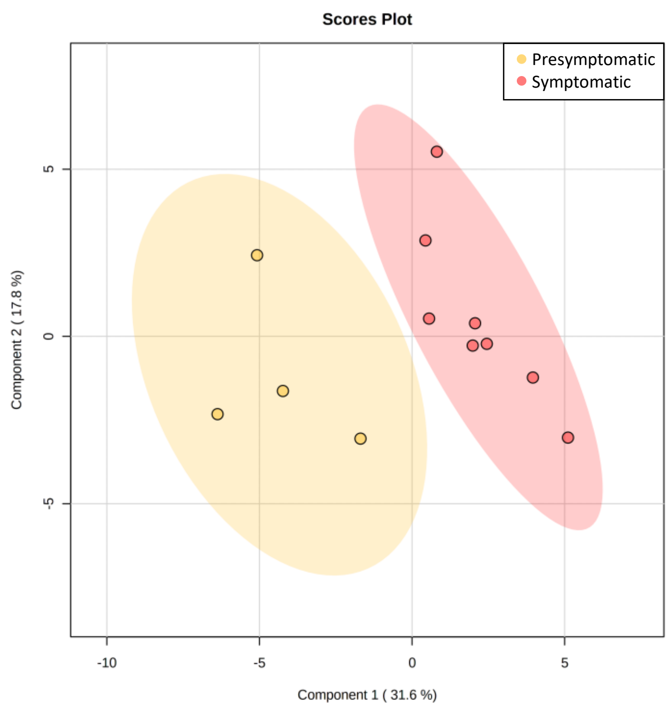

Fig. S6. Partial Least Squares Discriminant Analysis (PLS-DA)
PLSDA clustering show 2D score plot for metabolomic sample of HD serum of presymptomatic vs symptomatic. Figure generated using MetaboAnalyst 5.0- https://www.metaboanalyst.ca/.

Disease Models & Mechanisms • Supplementary information

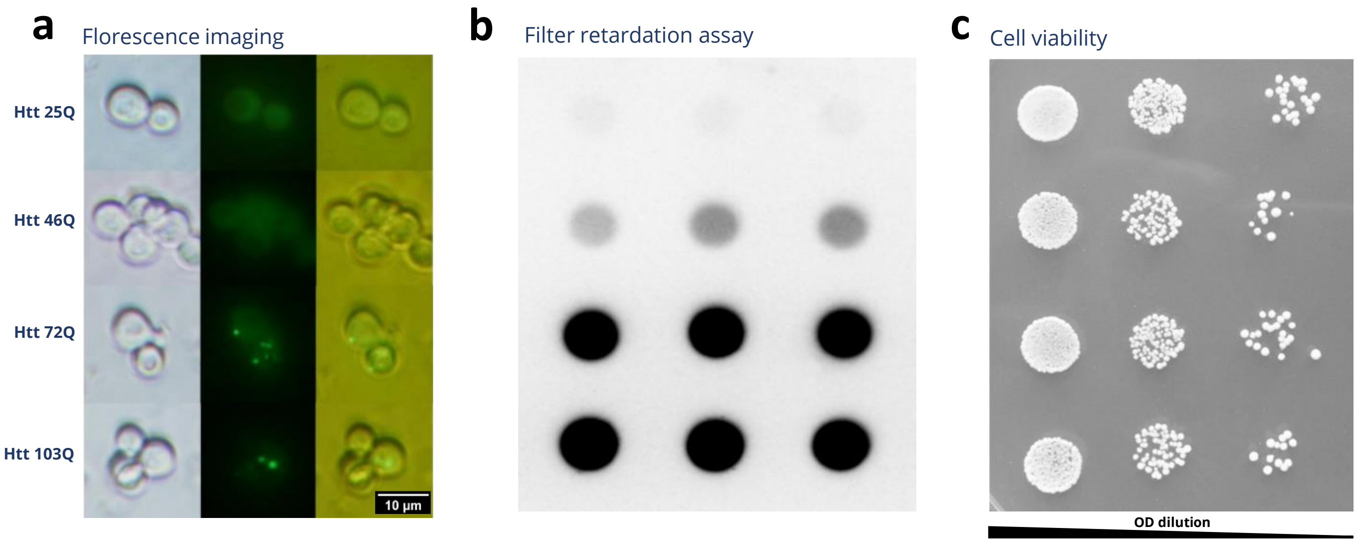

Fig. S7. Yeast model of HD.

(a) Yeast strain BY4741 transformed with the Htt 25Q, 46Q, 72Q and 103Q plasmid tagged with GFP showing aggregation as foci. Intense foci is observed in case of 72Q and 103Q where as it is more diffused in 46Q transformed cells, 25Q serving as the control (n=3). (b) Filter retardation assay (Dot blot assay) of these PolyQ transformed yeast show more aggregation in 72Q and 103Q compared to 46Q or 25Q (n=3). (c) The cell viability was determined with spot test showing PolyQ transformation have no detrimental effect on cell growing with increase in PolyQ length (n=3).

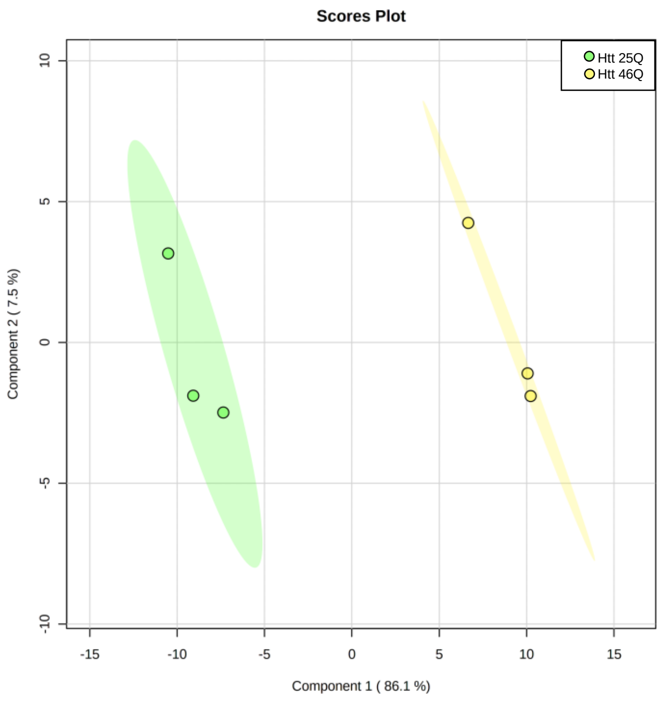

Fig. S8. Partial Least Squares Discriminant Analysis (PLS-DA)

PLSDA clustering show 2D score plot for metabolomic sample (negative mode) of Htt 25Q vs 46Q. Figure generated using MetaboAnalyst 5.0- https://www.metaboanalyst.ca/.

Disease Models & Mechanisms • Supplementary information

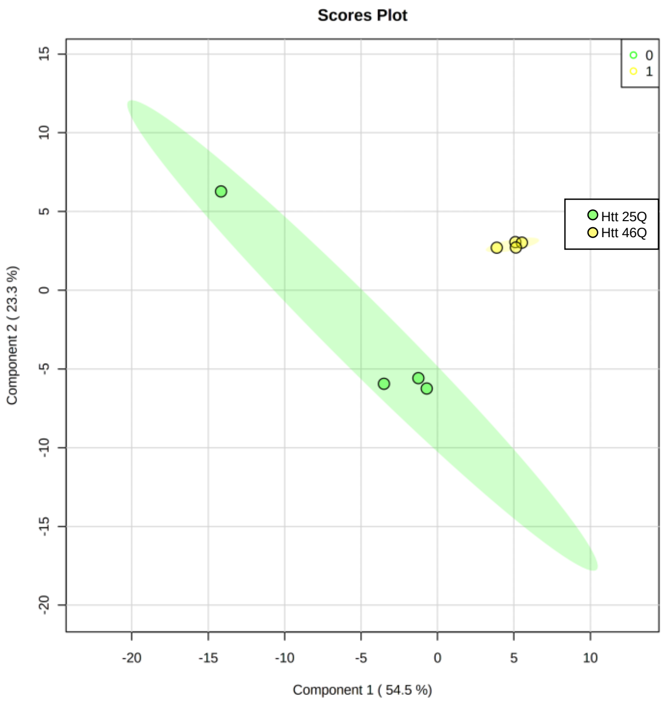

Fig. S9. Partial Least Squares Discriminant Analysis (PLS-DA)

PLSDA clustering show 2D score plot for metabolomic sample (positive mode) of Htt 25Q vs 46. Figures generated using MetaboAnalyst 5.0-https://www.metaboanalyst.ca/.

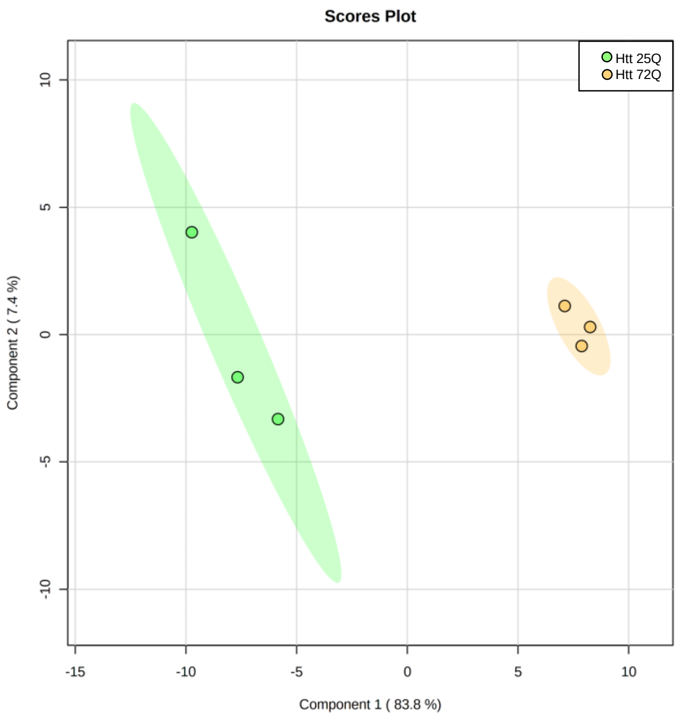

Fig. S10. Partial Least Squares Discriminant Analysis (PLS-DA)

PLSDA clustering show 2D score plot for metabolomic sample (negative mode) of Htt 25Q vs 72Q. Figure generated using MetaboAnalyst 5.0- https://www.metaboanalyst.ca/.

Disease Models & Mechanisms • Supplementary information

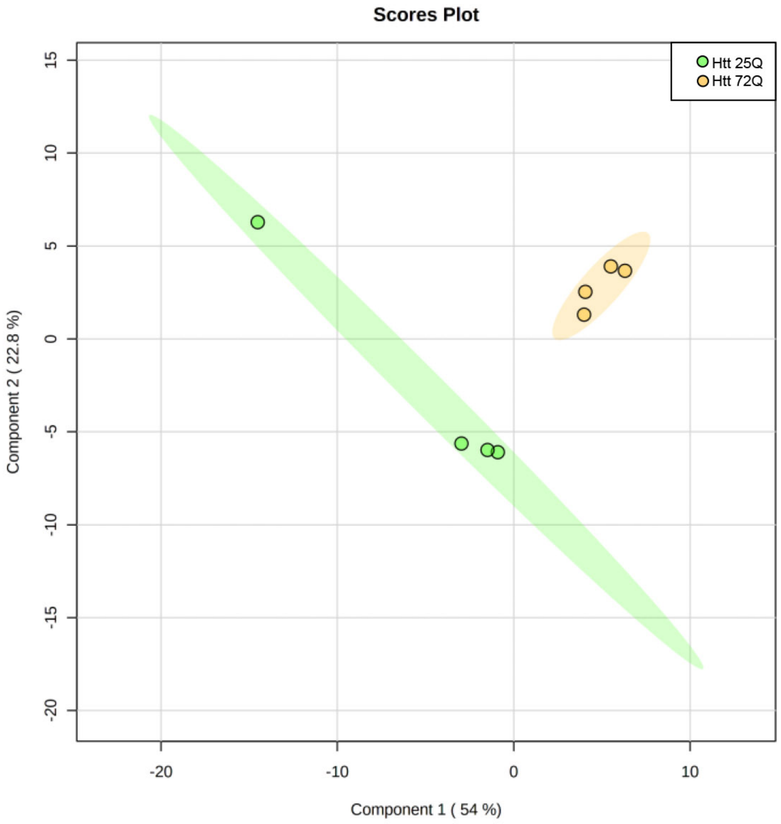

Fig. S11. Partial Least Squares Discriminant Analysis (PLS-DA)
PLSDA clustering show 2D score plot for metabolomic sample (positive mode) of Htt 25Q vs 72Q. Figure generated using MetaboAnalyst 5.0-https://www.metaboanalyst.ca/.

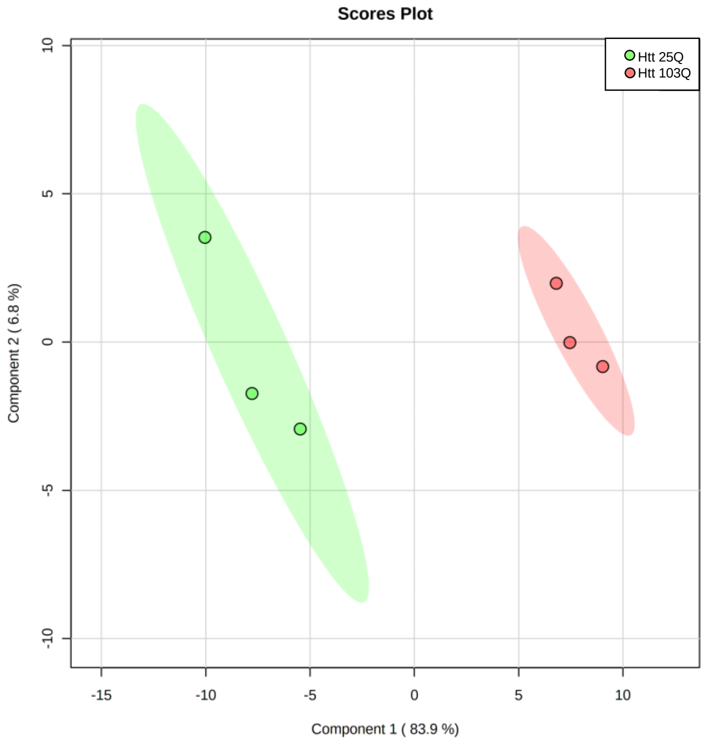

Fig. S12. Partial Least Squares Discriminant Analysis (PLS-DA)
PLSDA clustering show 2D score plot for metabolomic sample (negative mode) of Htt 25Q vs 103Q. Figure generated using MetaboAnalyst 5.0- https://www.metaboanalyst.ca/.

Disease Models & Mechanisms • Supplementary information

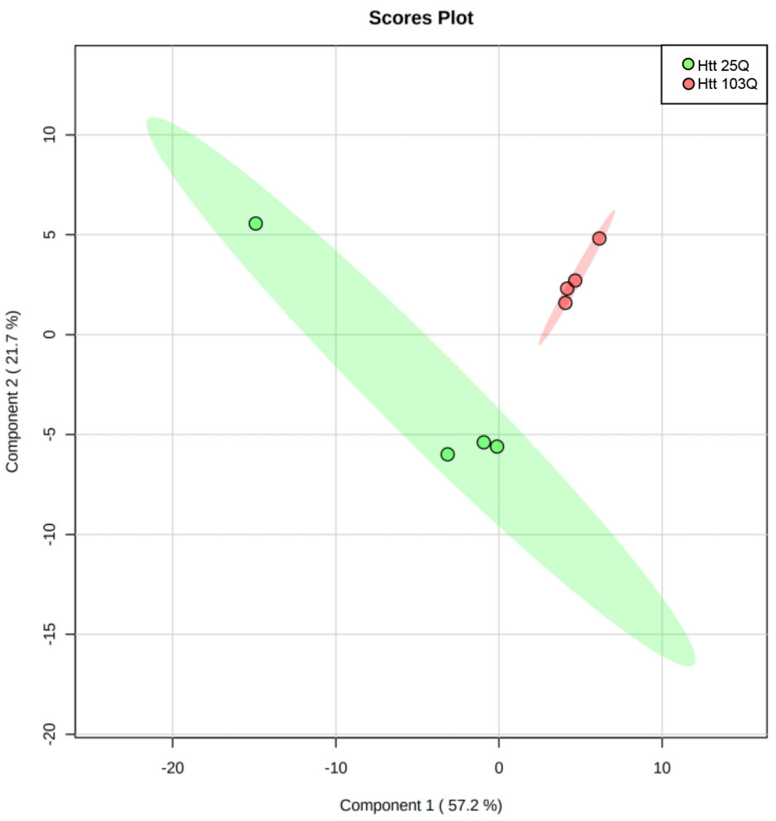

Fig. S13. Partial Least Squares Discriminant Analysis (PLS-DA)

PLSDA clustering show 2D score plot for metabolomic sample (negative mode) of Htt 25Q vs 103Q. Figures generated using MetaboAnalyst 5.0- https://www.metaboanalyst.ca/.

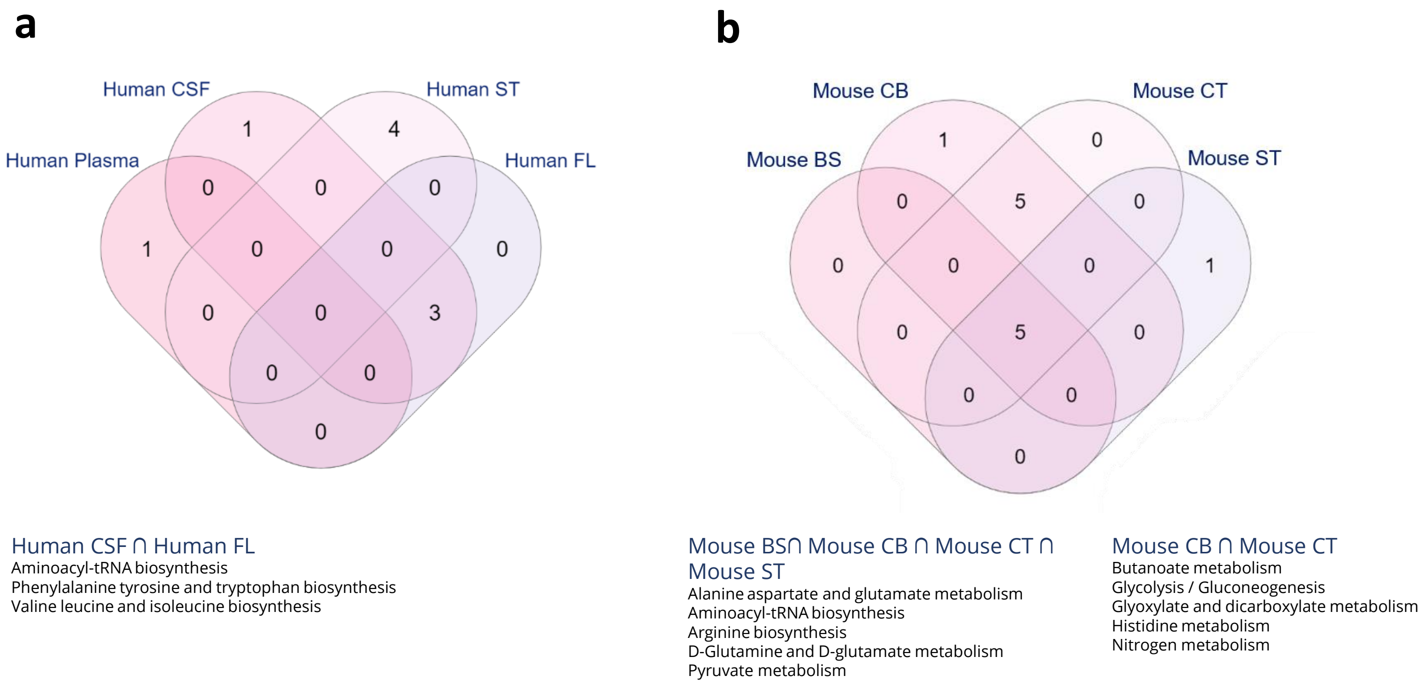

Fig. S14. Enriched pathway comparison (Metabolomics)

Illustration of common pathways between samples of (A) HD patient and (B) Mouse model of HD. The intersection represents common pathways enriched among samples. (The comparative Venn diagram illustration was generated using online tool-https:// www.molbiotools.com/listcompare.html).

Disease Models & Mechanisms • Supplementary information

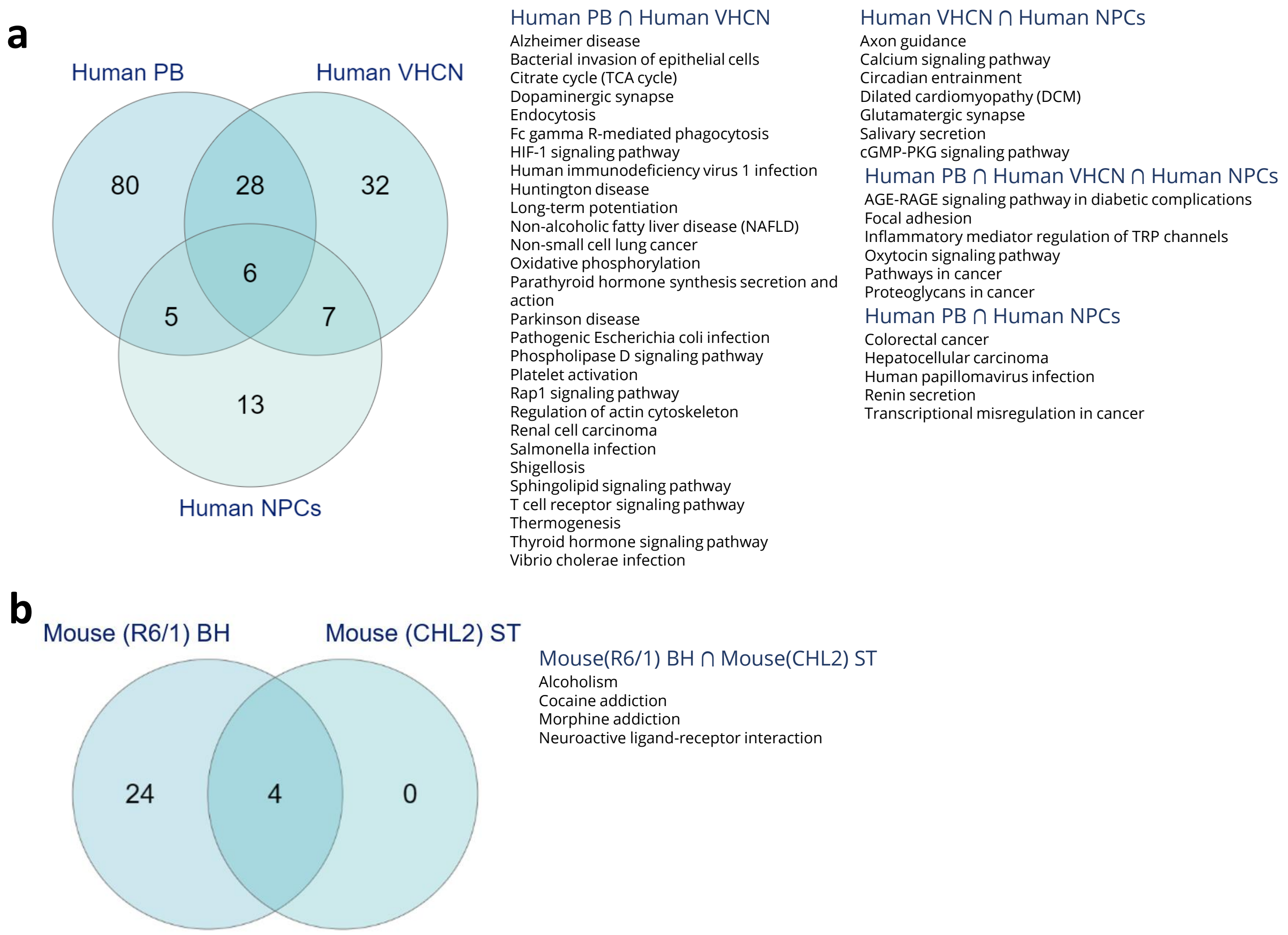

Fig. S15. Enriched pathway comparison (Transcriptomics)

Illustration of common pathways enriched between (A) HD patient datasets and (B) datasets of Mouse model of HD. Intersections representing common pathways deregulate among samples. (The Venn diagrams were created using https://www.molbiotools.com/ listcompare.html an online tool).

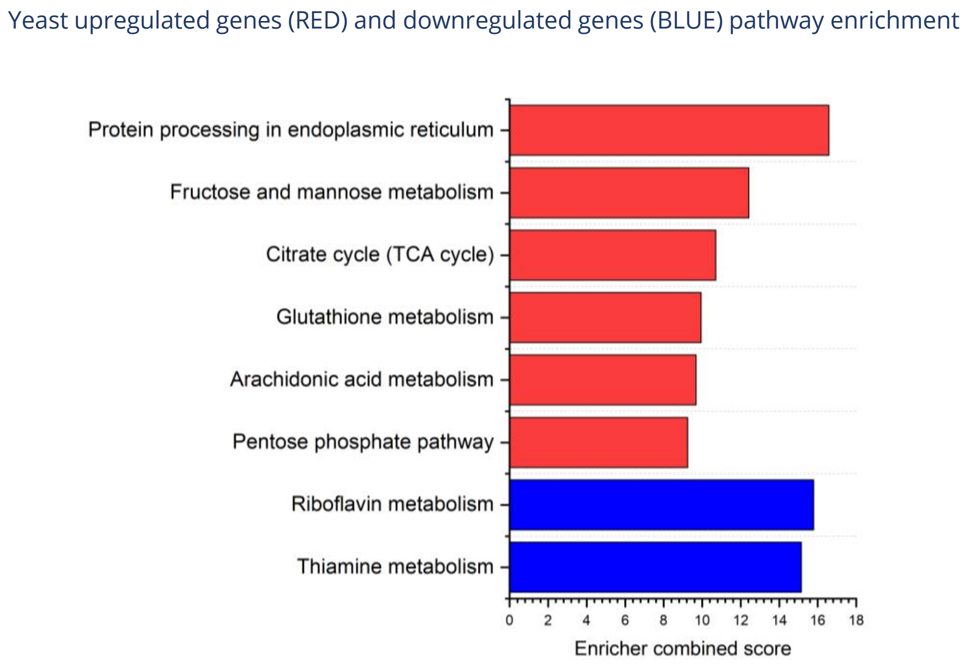

Fig. S16. Enriched Pathway

The chart illustrates results from the enrichment analysis of the upregulated (shown in red) and down regulated (shown in blue) genes in the disease condition. The pathways are sorted based on their combined score ranking. (Histograms plotted using OriginPro 8.5).

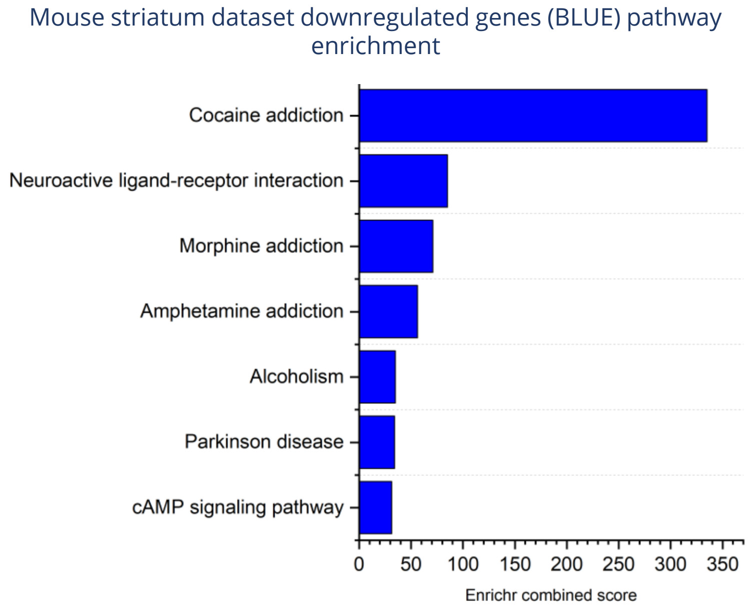

Fig. S17. Enriched Pathway

The chart illustrates results from the enrichment analysis of the negatively deregulated (downregulated) genes in the disease condition. The pathways are sorted based on their combined score ranking. (Histograms plotted using OriginPro 8.5).

Disease Models & Mechanisms • Supplementary information

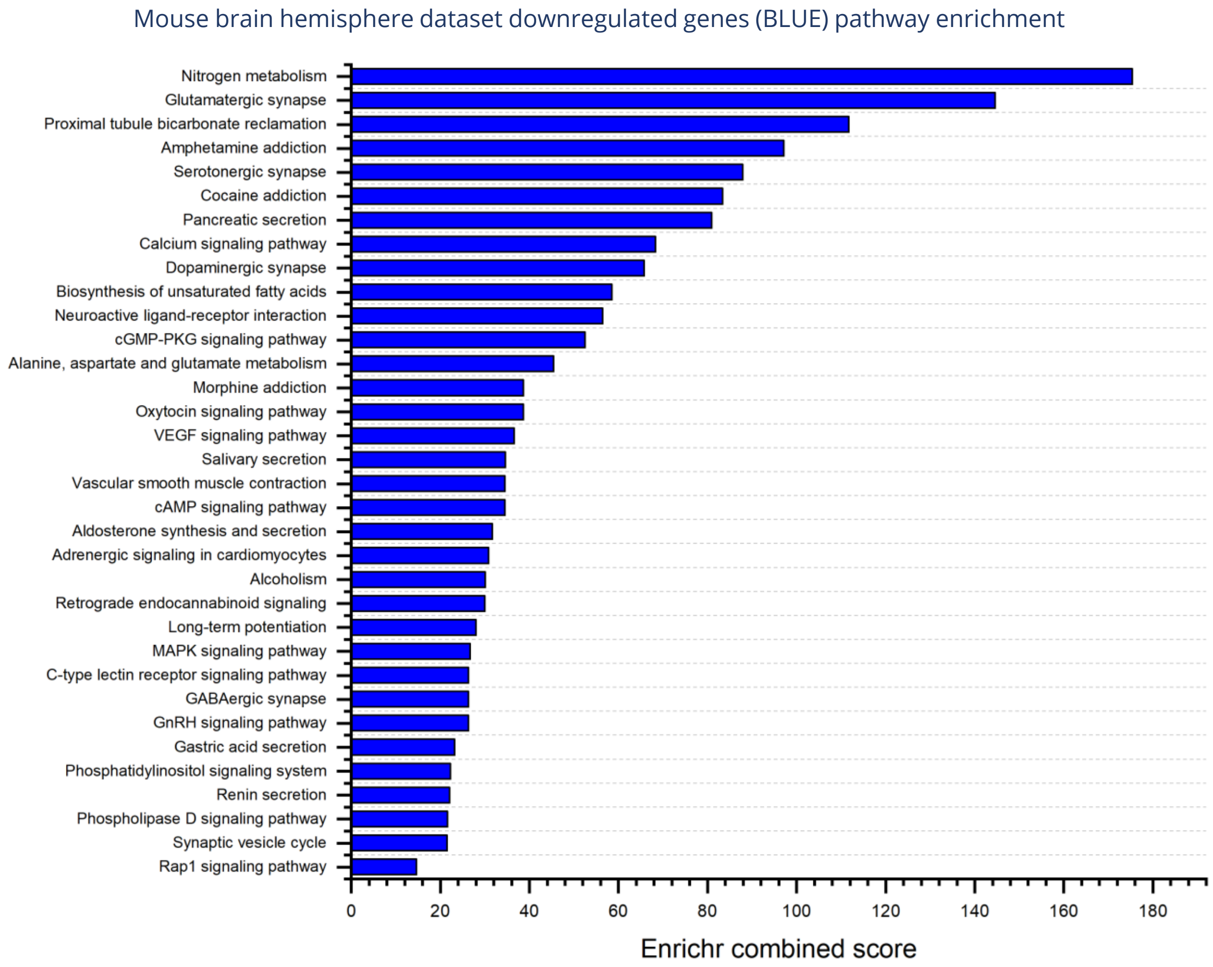

Fig. S18. Enriched Pathway
The chart illustrates results from the enrichment analysis of the negatively deregulated (down regulated) genes in the disease condition. The pathways are sorted based on their combined score ranking. (Histograms plotted using OriginPro 8.5).

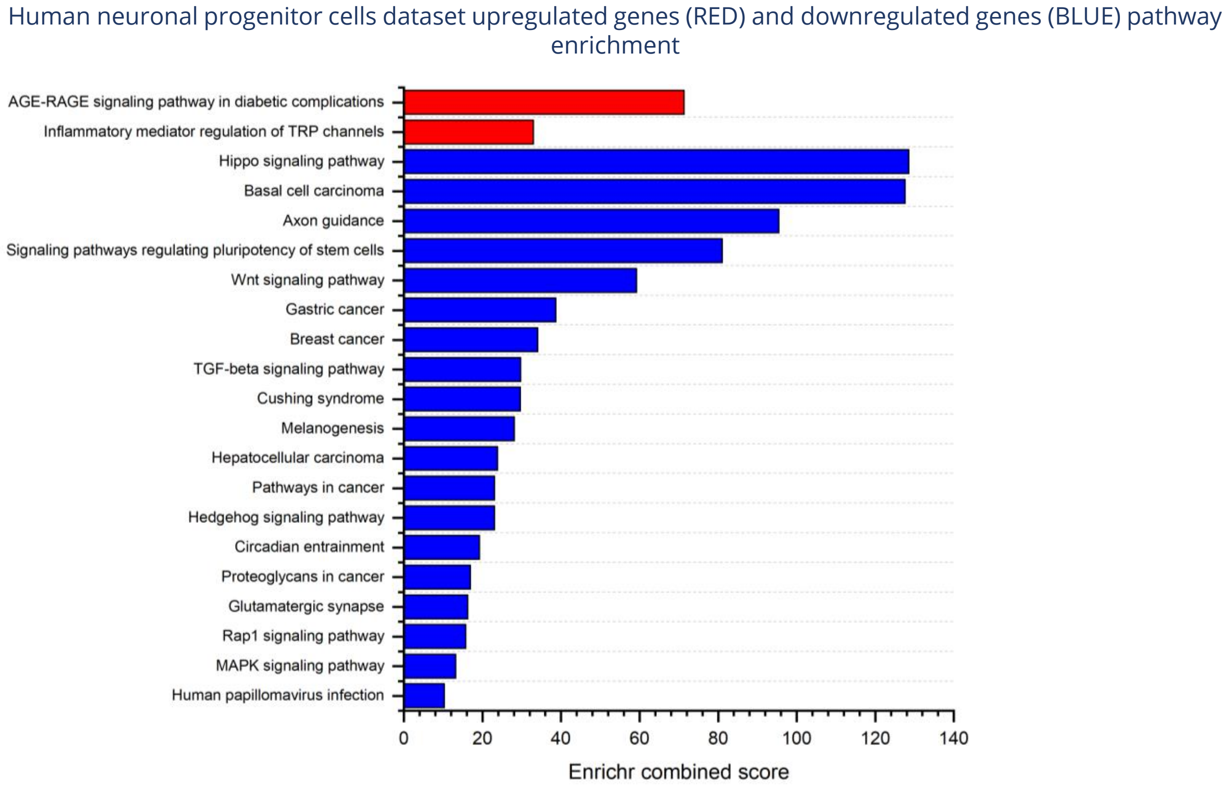

Fig. S19. Enriched Pathway
The chart illustrates results from the enrichment analysis of the upregulated (shown in red) and down regulated (shown in blue) genes in the disease condition. The pathways are sorted based on their combined score ranking. (Histograms plotted using OriginPro 8.5).

Disease Models & Mechanisms • Supplementary information

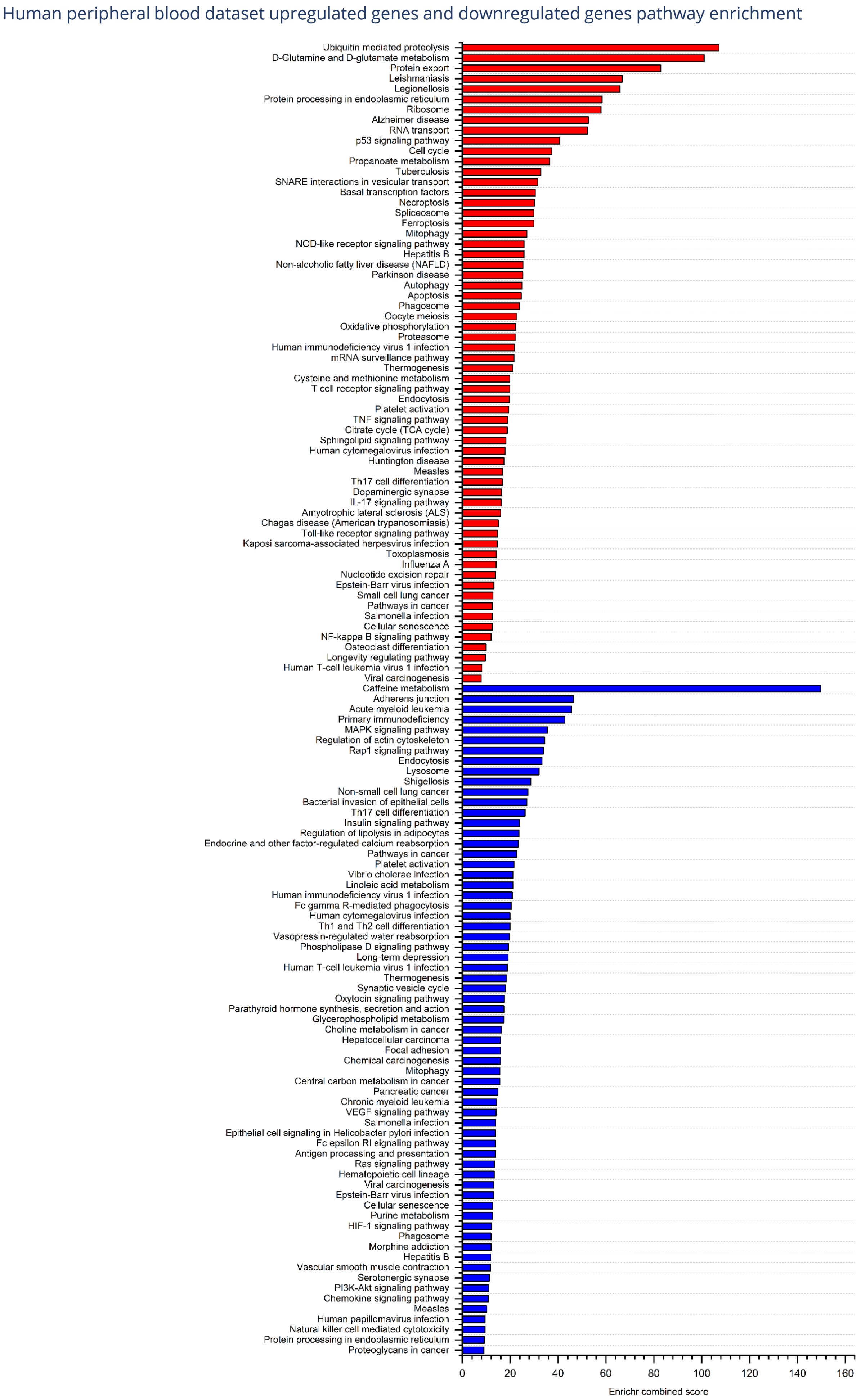

Fig. S20. Enriched Pathway

The chart illustrates results from the enrichment analysis of the upregulated (shown in red) and down regulated (shown in blue) genes in the disease condition. The pathways are sorted based on their combined score ranking. (Histograms plotted using OriginPro 8.5).

Disease Models & Mechanisms • Supplementary information

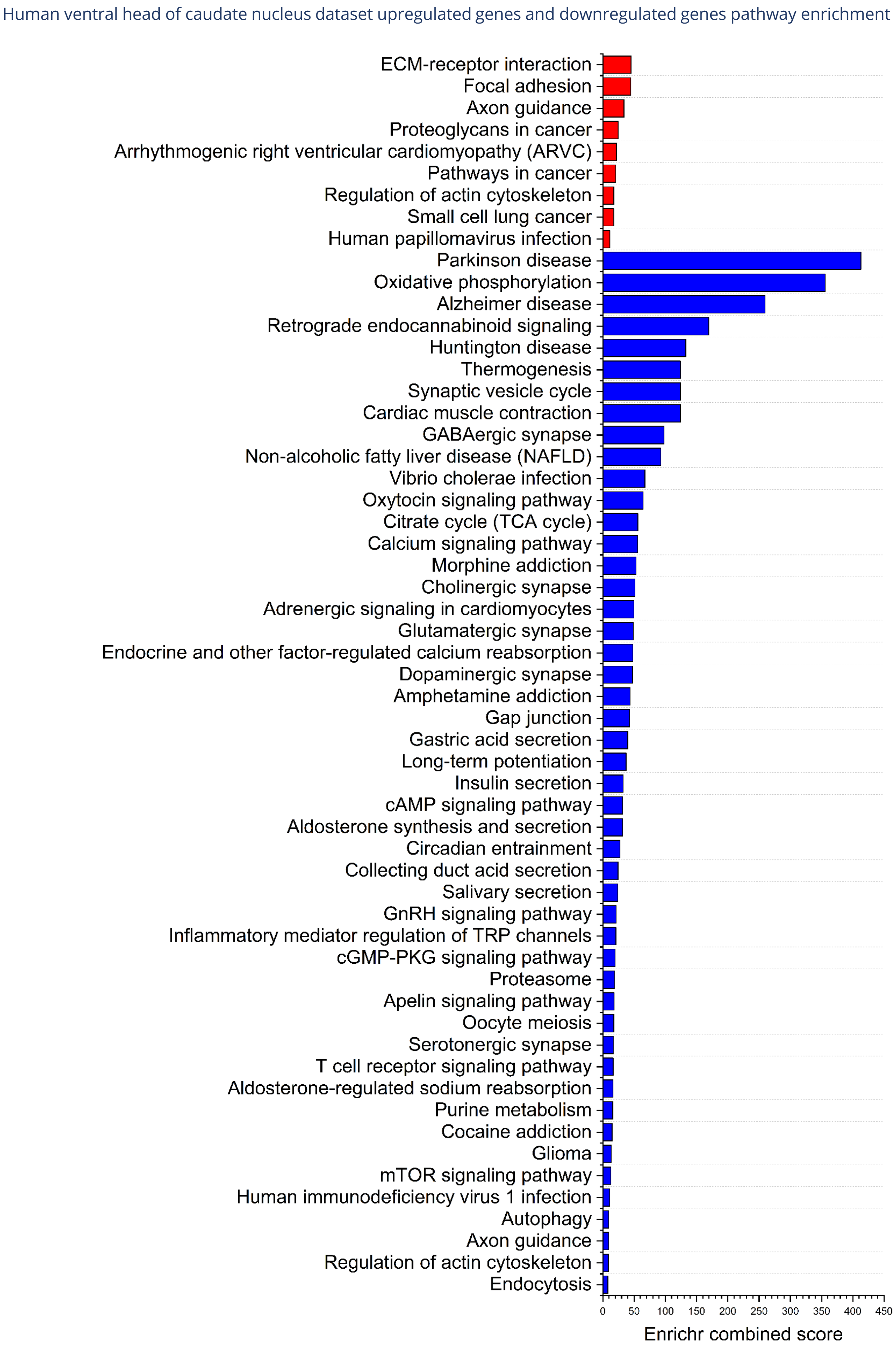

Fig. S21. Enriched Pathway

The chart illustrates results from the enrichment analysis of the upregulated (shown in red) and down regulated (shown in blue) genes in the disease condition. The pathways are sorted based on their combined score ranking. (Histograms plotted using OriginPro 8.5).

Disease Models & Mechanisms • Supplementary information

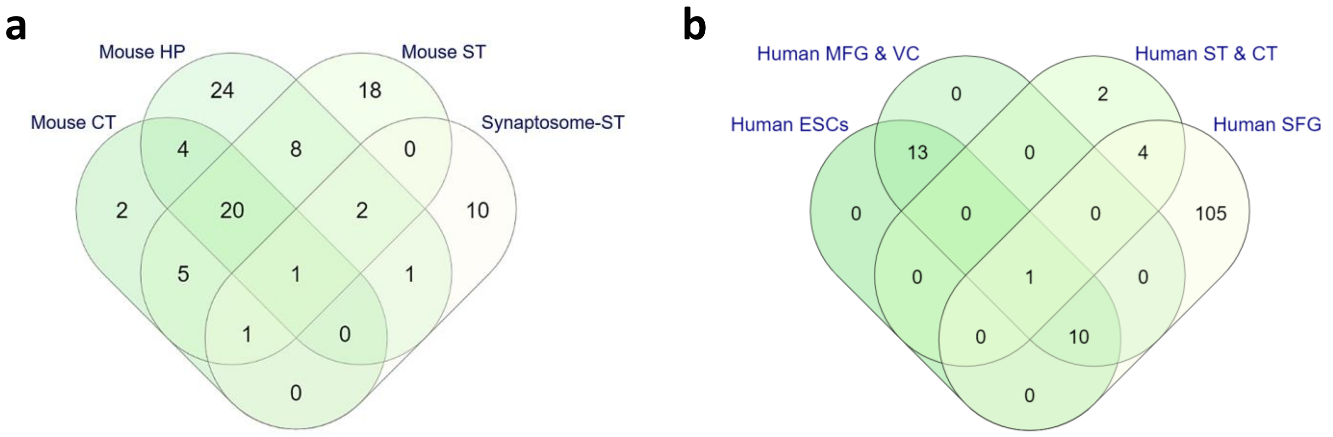

Mouse CT ∩ Mouse HP ∩ Mouse ST
Adrenergic signaling in cardiomyocytes
Alcoholism
Aldosterone synthesis and secretion
Amphetamine addiction
Amyotrophic lateral sclerosis (ALS)
Apelin signaling pathway
Arginine and proline metabolism
Calcium signaling pathway
Cholinergic synapse
Circadian entrainment
Cocaine addiction
Dopaminergic synapse
Fatty acid degradation
Glutamatergic synapse
Long-term potentiation
Morphine addiction
Oxytocin signaling pathway
Proteasome
Protein processing in endoplasmic reticulum
cAMP signaling pathway

Mouse HP ∩ Synaptosome-ST
Cardiac muscle contraction

Mouse CT ∩ Mouse HP ∩ Mouse ST ∩ Synaptosome-ST
Retrograde endocannabinoid signaling

Mouse HP ∩ Mouse ST
Arginine biosynthesis
Choline metabolism in cancer
GnRH signaling pathway
Phosphatidylinositol signaling system
Salivary secretion
Serotonergic synapse
Tight junction
Vascular smooth muscle contraction

Mouse CT ∩ Mouse HP
Endocytosis
GABAergic synapse
Tryptophan metabolism
beta-Alanine metabolism

Mouse CT ∩ Mouse ST
Axon guidance
Epstein-Barr virus infection
Purine metabolism
RNA transport
Renin secretion

Mouse HP ∩ Mouse ST ∩ Synaptosome-ST
Alzheimer disease
Pyruvate metabolism

Mouse CT ∩ Mouse ST ∩ Synaptosome-ST
Valine leucine and isoleucine degradation

Human ESCs ∩ Human MFG & VC ∩ Human ST & CT ∩ Human SFG
Fructose and mannose metabolism

Human ESCs ∩ Human MFG & VC
African trypanosomiasis
Antigen processing and presentation
Apoptosis
Fluid shear stress and atherosclerosis
Influenza A
Legionellosis
Malaria
Measles
Phagosome
Spliceosome
Synaptic vesicle cycle
Toxoplasmosis
Vibrio cholerae infection

Human ESCs ∩ Human MFG & VC ∩ Human SFG
Bacterial invasion of epithelial cells
Endocytosis
Estrogen signaling pathway
Gap junction
Glycolysis / Gluconeogenesis
HIF-1 signaling pathway
Longevity regulating pathway
Pathogenic Escherichia coli infection
Platelet activation
Proteasome

Human ST & CT ∩ Human SFG
Citrate cycle (TCA cycle)
Glutathione metabolism
Glyoxylate and dicarboxylate metabolism
Pentose phosphate pathway

Fig. S22. Enriched pathway comparison (Proteomics)

Illustration of common pathways between samples of (a) Mouse model of HD and (b) HD patient. The intersection represents common pathways enriched among samples. (Venn diagrams for comparison was created using an online tool-https://www.molbiotools. com/ listcompare.html).

Disease Models & Mechanisms • Supplementary information

Table S1. Data Summary table

Details on all the datasets of transcriptomics, proteomics and metabolomics used in the study. (Table made in Microsoft Excel).

Organism	Sample	References	Paper title
**Transcriptomics**			
Saccharomyces cerevisiae	Whole cell	GSE18644	Functional gene expression profiling in yeast implicates translational dysfunction in mutant huntingtin toxicity
Mus musculus	Brain hemisphere	GSE3621	Brain gene expression correlates with changes in behavior in the R6/1 mouse model of Huntington's disease
	Striatum	GSE10202	Mutant huntingtin's effects on striatal gene expression in Mouse recapitulate changes observed in human Huntington's disease brain and do not differ with mutant huntingtin length or wild-type huntingtin dosage
Homo sapiens	Ventral head of caudate nucleus	GSE26927	Selection of novel reference genes for use in the human central nervous system: a BrainNet Europe Study
	Peripheral blood sample	GSE1751	Genome-wide expression profiling of human blood reveals biomarkers for Huntington's disease
	iPSCs & derived NPCs	GSE93767	Reversal of Phenotypic Abnormalities by CRISPR/Cas9-Mediated Gene Correction in Huntington Disease Patient-Derived Induced Pluripotent Stem Cells
**Proteomics**			
Mus musculus	Striatum<br>Cortex<br>Hippocampus	PXD008099	Integrative Characterization of the R6/2 Mouse Model of Huntington's Disease Reveals Dysfunctional Astrocyte Metabolism
	Synaptosomes from Striatum and Cortex	https://doi.org/10.1016/j.nbd.2020.104950	Protein changes in synaptosomes of Huntington's disease knock-in Mouse are dependent on age and brain region
Homo sapiens	Superior frontal gyrus tissue	https://doi.org/10.1021/acs.jproteome.6b00448	Quantitative Proteomic Analysis Reveals Similarities between Huntington's Disease (HD) and Huntington's Disease-Like 2 (HDL2) Human Brains
	Middle frontal gyrus and the visual cortex	10.3233/JHD-120044	Proteomic Analysis of the Human Brain in Huntington's Disease Indicates Pathogenesis by Molecular Processes Linked to other Neurodegenerative Diseases and to Type-2 Diabetes
	Striatum and cortex	10.1016/j.freeradbiomed.2008.05.014	Proteomic and oxidative stress analysis in human brain samples of Huntington disease
	Embryonic Stem Cells	https://doi.org/10.1021/pr500649m	Proteomics of Huntington's Disease-Affected Human Embryonic Stem Cells Reveals an Evolving Pathology Involving Mitochondrial Dysfunction and Metabolic Disturbances
**Metabolomics**			
Saccharomyces cerevisiae	Whole cell	https://doi.org/10.1021/pr900734g	Probing the Metabolic Aberrations Underlying Mutant Huntingtin Toxicity in Yeast and Assessing Their Degree of Preservation in Humans and Mouse
Mus musculus	Brain region	https://doi.org/10.1021/pr050244o	Metabolic Characterization of the R6/2 Transgenic Mouse Model of Huntington's Disease by High-Resolution MAS 1H NMR Spectroscopy
Homo sapiens	Blood plasma	https://doi.org/10.1002/acn3.214	A systems-level "misunderstanding": the plasma metabolome in Huntington's disease
	Cerebrospinal fluid	https://doi.org/10.1038/s41598-019-40186-5	Alterations in the tyrosine and phenylalanine pathways revealed by biochemical profiling in cerebrospinal fluid of Huntington's disease subjects
	Frontal lobe and striatum	https://doi.org/10.1021/acs.jproteome.6b00049	Novel Metabolite Biomarkers of Huntington's Disease As Detected by High-Resolution Mass Spectrometry
	Frontal lobe and striatum	https://doi.org/10.1016/j.bbadis.2018.04.012	Targeted biochemical profiling of brain from Huntington's disease patients reveals novel metabolic pathways of interest

Table S2. Patient Data

Details on patient Demographics and clinical information. The patients information has been acquired and modified for previously described table since most of the patients are from the previous cohort (Thota et al., 2021).

Patient	Family	Relation	Age at examination	Gender	CAG repeats	
Presymptomatic_1	Family B	Daughter of Symptomatic_8	18	F	45	17
Presymptomatic_2	Family B	Sibling (Patient)	39	F	44	17
Presymptomatic_3	Family B	Sibling (Patient)	25	M	52	17
Presymptomatic_4	Family B	Sibling (Patient)	27	M	47	17
Presymptomatic_5	Family I	Patient	28	F	44	15
Symptomatic_1	Family H	Patient	40	M	44	17
Symptomatic_2	Family G	Patient	43	M	43	17
Symptomatic_3	Family F	Patient	22	M	62	20
Symptomatic_4	Family E	Patient	27	F	56	17
Symptomatic_5	Family D	Patient	26	M	55	17
Symptomatic_6	Family C	Sibling (Patient)	41	F	46	17
Symptomatic_7	Family C	Sibling (Patient)	42	M	47	17
Symptomatic_8	Family B	Sibling (Patient)	32	F	51	17
Symptomatic_9	Family A	Patient	51	M	43	17
Symptomatic_10	Family J	Patient	50	F	44	21
Symptomatic_11	Family K	Patient	26	F	57	17

Disease Models & Mechanisms • Supplementary information

Table S3. 89 Detected metabolites and Multiple reaction monitoring (MRM) method of HD patient samples analysed in positive ionization mode.

Compound Name	ISD	Precursor ion	Product ion	Dwell	Frag(V)	CE(V)	Polarity
1-Methyl-Histidine	FALSE	170.0924	124	3	380	20	POSITIVE
2-Aminobutyraldehyde	FALSE	88.0757	70.1	3	380	9	POSITIVE
2-Aminoheptanoic acid	FALSE	146.1176	100.1	3	380	5	POSITIVE
2-Aminooctanoic acid	FALSE	160.1332	55.3	3	380	21	POSITIVE
2-Hydroxypyridine	FALSE	96.0444	78.1	3	380	17	POSITIVE
2-Methyl glutamic acid	FALSE	162.0761	116.1	3	380	9	POSITIVE
2-Methylbutyroylcarnitine	FALSE	246.17	187	3	380	15	POSITIVE
4 Acetamidobutanoic acid	FALSE	146.08	86.1	3	380	9	POSITIVE
4 Hydroxybutyric acid	FALSE	105.0546	45	3	380	5	POSITIVE
4-Coumarate	FALSE	165.0446	119	3	380	10	POSITIVE
5-Hydroxyindoleacetic acid	FALSE	192.0655	146	3	380	10	POSITIVE
5-oxo-proline	FALSE	130.0499	84	3	380	10	POSITIVE
Acetyl Carnitine	FALSE	204.123	85	3	380	9	POSITIVE
Acetylcholine	FALSE	146.1176	43.02	3	380	10	POSITIVE
Adenosine	FALSE	268.1	137.1	3	380	12	POSITIVE
Allo-Threonine	FALSE	120.0655	74.1	3	380	5	POSITIVE
Aminobutyric acid	FALSE	104.0706	44.1	3	380	9	POSITIVE
Arginine	FALSE	175.119	70.1	3	380	25	POSITIVE
betaine aldehyde	FALSE	103.0992	58	3	380	21	POSITIVE
Betaine	FALSE	118.0863	59.2	3	380	17	POSITIVE
Carnitine	FALSE	162.1125	60.1	3	380	12	POSITIVE
Choline	FALSE	105.1148	45	3	380	21	POSITIVE
citrulline	FALSE	176.103	159	3	380	5	POSITIVE
Creatinine	FALSE	114.0662	44	3	380	17	POSITIVE
Cytosine	FALSE	112.1	95.1	3	380	17	POSITIVE
Deoxy carnitine	FALSE	146.1176	60.1	3	380	13	POSITIVE
Dimethyl Arginine	FALSE	203.1503	70.1	3	380	29	POSITIVE
DL-Pipecolic acid	FALSE	130.0863	84	3	380	18	POSITIVE
Erythrono-1,4-lactone	FALSE	119.11	91.1	3	380	10	POSITIVE
Ethylmalonate/Methyl Malonate	FALSE	133.0495	115.04	3	380	10	POSITIVE
Glucosamine/Galactosamine	FALSE	180.0866	162	3	380	5	POSITIVE
Glutamic acid	FALSE	148.0604	84	3	380	17	POSITIVE
Glutamyl alanine	FALSE	219.0975	84.1	3	380	21	POSITIVE
Glycine	FALSE	76.0393	30	3	380	9	POSITIVE
Gly-Gly	FALSE	133.0608	30	3	380	20	POSITIVE
Gly-Leu	FALSE	189.1234	130	3	380	10	POSITIVE
Guanidine acetic acid	FALSE	118.0611	43.1	3	380	40	POSITIVE
Homoserine	FALSE	120.0655	74.1	3	380	9	POSITIVE
Hypoxanthine	FALSE	137	119	3	380	21	POSITIVE
Isobutyryl Carnitine	FALSE	232.1543	85	3	380	21	POSITIVE
Isoleucine	FALSE	132.1019	86.1	3	380	9	POSITIVE
Kynurenic acid	FALSE	190.0499	144.1	3	380	16	POSITIVE
Kynurenine	FALSE	209.0921	192	3	380	5	POSITIVE
L-anthranilic acid	FALSE	139	121	3	380	12	POSITIVE
L-Creatinine	FALSE	115.1	45.1	3	380	10	POSITIVE
L-Thymine	FALSE	131.1	113.9	3	380	15	POSITIVE
Lysine	FALSE	147.1128	130.2	3	380	5	POSITIVE
Methylglutaric acid	FALSE	147.0652	55.05423	3	380	10	POSITIVE
Myristoleic acid	FALSE	227.2006	43.1	3	380	25	POSITIVE
N, N Dimethyl Glycine/Methyl alanin	FALSE	104.0706	58.1	3	380	12	POSITIVE
N, N Dimethyl Glycine	FALSE	104.0706	44	3	380	48	POSITIVE
N,N Dimethyl Glycine/Methyl alanine	FALSE	104.0706	58.1	3	380	9	POSITIVE
N-Acetyl Lysine	FALSE	189.1234	84.1	3	380	24	POSITIVE
N-acetylalanine	FALSE	132.0655	44.1	3	380	20	POSITIVE
N-acetyl-glutamine	FALSE	189.087	130	3	380	17	POSITIVE
N-Acetylornithine	FALSE	175.1077	70.1	3	380	29	POSITIVE
Nicotinamide	FALSE	123.0553	80.1	3	380	20	POSITIVE
Octonyl Carnitine	FALSE	288.2	85	3	380	21	POSITIVE
Oleic acid	FALSE	283.27	89.2	3	380	13	POSITIVE
Phenyl alanine	FALSE	166.0963	120.1	3	380	13	POSITIVE
Proline	FALSE	116.0706	70.1	3	380	17	POSITIVE
Propeonyl Carnitine	FALSE	218.1387	85	3	380	17	POSITIVE
purine	FALSE	121.0509	94	3	380	25	POSITIVE
Sarcosine/Alanine	FALSE	90.055	72.1	3	380	9	POSITIVE
Serine	FALSE	106.0499	60.1	3	380	9	POSITIVE
Spermidine	FALSE	146.1652	72.1	3	380	17	POSITIVE
Tryptophan	FALSE	205.1	146.1	3	380	10	POSITIVE
Tyrosine	FALSE	182.0812	136.1	3	380	9	POSITIVE
Urea	FALSE	61.0396	44	3	380	10	POSITIVE
Valine	FALSE	118.0863	72.2	3	380	9	POSITIVE
Xanthine	FALSE	153.04	110	3	380	21	POSITIVE
4-Methyl-2-oxo pentanoic acid	FALSE	131.0703	71	3	380	10	POSITIVE
Adenine	FALSE	136.6	119	3	380	10	POSITIVE
Asparagine	FALSE	133.0608	74	3	380	13	POSITIVE
Cystathionine	FALSE	223.0747	134.1	3	380	8	POSITIVE
Cystine	FALSE	241.03	152	3	380	9	POSITIVE
Glutamine	FALSE	147.0764	130.1	3	380	5	POSITIVE
Glycerol 3-phosphate	FALSE	173.02	132	3	380	5	POSITIVE
Heptanoyl Carnitine	FALSE	260.2	201.2	3	380	9	POSITIVE
Histamine	FALSE	112.0869	95.1	3	380	13	POSITIVE
Histidine	FALSE	156.0768	110.1	3	380	9	POSITIVE
Isovaleryl Carnitine	FALSE	246.2	85	3	380	17	POSITIVE
Myo-Inositol	FALSE	181.07	139.9	3	380	5	POSITIVE
N, N Dimethyl Glycine/Methyl alanine	FALSE	104.0706	58.1	3	380	12	POSITIVE
N-acetyl Glucosamine 6 Phos	FALSE	302.0635	284.1	3	380	5	POSITIVE
Palmitic acid	FALSE	257	57	3	380	40	POSITIVE
Quinic acid	FALSE	193.0707	111.04	3	380	10	POSITIVE
Sarcosine/Alainine	FALSE	90.055	44.1	3	380	20	POSITIVE
Uracil	FALSE	113	72	3	380	4	POSITIVE

Table S4. 109 Detected metabolites and Multiple reaction monitoring (MRM) method of Yeast samples analysed in positive ionization mode.

Compound Name	ISD	Precursor ion	Product ion	Dwell	Frag(V)	CE(V)	Polarity
1-Stearoyl-sn-glycero-3-phosphocholine	FALSE	524.3711	184.1	3	380	25	POSITIVE
2-Aminobutyraldehyde	FALSE	88.0757	70.1	3	380	9	POSITIVE
2-Hydroxypyridine	FALSE	96.0444	78.1	3	380	17	POSITIVE
2-Methyl glutamic acid	FALSE	162.0761	116.1	3	380	9	POSITIVE
3-phospho-serine	FALSE	186.0162	88	3	380	12	POSITIVE
4 Acetamidobutanoic acid	FALSE	146.08	86.1	3	380	9	POSITIVE
4 Hydroxybutyric acid	FALSE	105.0546	45	3	380	5	POSITIVE
4-Coumarate	FALSE	165.0446	119	3	380	10	POSITIVE
4-Methyl-2-oxo pentanoic acid	FALSE	131.0703	71	3	380	10	POSITIVE
5-Hydroxyindoleacetic acid	FALSE	192.0655	146	3	380	10	POSITIVE
5-oxo-proline	FALSE	130.0499	84	3	380	10	POSITIVE
Acetyl Carnitine	FALSE	204.123	85	3	380	9	POSITIVE
Acetylcholine	FALSE	146.1176	43.02	3	380	10	POSITIVE
Adenine	FALSE	136.6	119	3	380	10	POSITIVE
Adenosine	FALSE	268.1	137.1	3	380	12	POSITIVE
Allo-Threonine	FALSE	120.0655	74.1	3	380	5	POSITIVE
Aminobutyric acid	FALSE	104.0706	44.1	3	380	9	POSITIVE
Aminobytyric acid	FALSE	104.0706	69	3	380	22	POSITIVE
aminoimidazole carboxamide ribonucleotide	FALSE	339.07	110	3	380	32	POSITIVE
AMP	FALSE	348.0704	136	3	380	17	POSITIVE
Arginine	FALSE	175.119	70.1	3	380	25	POSITIVE
Asparagine	FALSE	133.0608	74	3	380	13	POSITIVE
Asparagine-15N2	FALSE	135	117	3	380	15	POSITIVE
Betaine	FALSE	118.0863	59.2	3	380	17	POSITIVE
betaine aldehyde	FALSE	103.0992	58	3	380	21	POSITIVE
Carnitine	FALSE	162.1125	60.1	3	380	12	POSITIVE
Choline	FALSE	105.1148	45	3	380	21	POSITIVE
citrulline	FALSE	176.103	159	3	380	5	POSITIVE
Creatinine	FALSE	114.0662	44	3	380	17	POSITIVE
Cystathionine	FALSE	223.0747	134.1	3	380	8	POSITIVE
Cysteine	FALSE	122.027	76	3	380	9	POSITIVE
Cystine	FALSE	241.03	152	3	380	9	POSITIVE
Cytidine 5 -Monophosphate (5-CMP)	FALSE	324.0591	112	3	380	9	POSITIVE
Cytosine	FALSE	112.1	95.1	3	380	17	POSITIVE
Deoxy carnitine	FALSE	146.1176	60.1	3	380	13	POSITIVE
dGMP	FALSE	348.0704	135	3	380	38	POSITIVE
Dimethyl Arginine	FALSE	203.1503	70.1	3	380	29	POSITIVE
DL-Pipecolic acid	FALSE	130.0863	84	3	380	18	POSITIVE
Erythrono-1,4-lactone	FALSE	119.11	91.1	3	380	10	POSITIVE
Ethylmalonate/Methyl Malonate	FALSE	133.0495	115.04	3	380	10	POSITIVE
Glucosamine/Galactosamine	FALSE	180.0866	162	3	380	5	POSITIVE
Glu-Glu	FALSE	277.103	84.04	3	380	10	POSITIVE
Glutamic acid	FALSE	148.0604	84	3	380	17	POSITIVE
Glutamine	FALSE	147.0764	130.1	3	380	5	POSITIVE
Glutamyl alanine	FALSE	219.0975	84.1	3	380	21	POSITIVE
Glutathione, Reduced (GSH)	FALSE	308.0911	179	3	380	9	POSITIVE
Glycerol 3-phosphate	FALSE	173.02	132	3	380	5	POSITIVE
Glycine	FALSE	76.0393	30	3	380	9	POSITIVE
Gly-Gly	FALSE	133.0608	30	3	380	20	POSITIVE
Gly-Leu	FALSE	189.1234	130	3	380	10	POSITIVE
GMP	FALSE	364	152	3	380	12	POSITIVE
GMP	FALSE	364.0653	152	3	380	13	POSITIVE
Guanidine acetic acid	FALSE	118.0611	43.1	3	380	40	POSITIVE
Guanosine	FALSE	284.0989	152	3	380	9	POSITIVE
Heptanoyl Carnitine	FALSE	260.2	201.2	3	380	9	POSITIVE
Histamine	FALSE	112.0869	95.1	3	380	13	POSITIVE
Histidine	FALSE	156.0768	110.1	3	380	9	POSITIVE
Homoserine	FALSE	120.0655	74.1	3	380	9	POSITIVE
Hydroxyglutaric acid	FALSE	132	45	3	380	30	POSITIVE
Hypoxanthine	FALSE	137	119	3	380	21	POSITIVE
Isoleucine	FALSE	132.1019	86.1	3	380	9	POSITIVE
Isovaleryl Carnitine	FALSE	246.2	85	3	380	17	POSITIVE
Kynurenic acid	FALSE	190.0499	144.1	3	380	16	POSITIVE
Kynurenine	FALSE	209.0921	192	3	380	5	POSITIVE
Lactic acid	FALSE	91.04	73	3	380	15	POSITIVE
L-anthranilic acid	FALSE	139	121	3	380	12	POSITIVE
L-Arginine	FALSE	177.1	159.1	3	380	10	POSITIVE
L-Thymine	FALSE	131.1	113.9	3	380	15	POSITIVE
Lysine	FALSE	147.1128	130.2	3	380	5	POSITIVE
Methyl Nicotinamide	FALSE	138.08	95.1	3	380	25	POSITIVE
Methylglutaric acid	FALSE	147.0652	55.05423	3	380	10	POSITIVE
Myo-Inositol	FALSE	181.07	139.9	3	380	5	POSITIVE
Myristoleic acid	FALSE	227.2006	43.1	3	380	25	POSITIVE
N, N Dimethyl Glycine	FALSE	104.0706	44	3	380	48	POSITIVE
N, N Dimethyl Glycine/Methyl alanin	FALSE	104.0706	58.1	3	380	12	POSITIVE
N, N Dimethyl Glycine/Methyl alanine	FALSE	104.0706	58.1	3	380	12	POSITIVE
N,N Dimethyl Glycine/Methyl alanine	FALSE	104.0706	58.1	3	380	9	POSITIVE
NAAG	FALSE	305.0979	84.1	3	380	41	POSITIVE
N-acetyl Glucosamine 6 Phos	FALSE	302.0635	284.1	3	380	5	POSITIVE
N-Acetyl Lysine	FALSE	189.1234	84.1	3	380	24	POSITIVE
N-acetylalanine	FALSE	132.0655	44.1	3	380	20	POSITIVE
N-acetyl-glutamine	FALSE	189.087	130	3	380	17	POSITIVE
N-Acetylornithine	FALSE	175.1077	70.1	3	380	29	POSITIVE
N-Acetylputrescine	FALSE	131.1179	114	3	380	12	POSITIVE
NAD	FALSE	664.1164	136.06	3	380	60	POSITIVE
N-carbamoyl-L-aspartate	FALSE	177.0506	74	3	380	19	POSITIVE
Nicotinamide	FALSE	123.0553	80.1	3	380	20	POSITIVE
Nicotinamide ribotide	FALSE	335	123	3	380	30	POSITIVE
Octonyl Carnitine	FALSE	288.2	85	3	380	21	POSITIVE
Oleic acid	FALSE	283.27	89.2	3	380	13	POSITIVE
Palmitic acid	FALSE	257	57	3	380	40	POSITIVE
Phenyl alanine	FALSE	166.0963	120.1	3	380	13	POSITIVE
Phospho Choline	FALSE	185.0811	99	3	380	37	POSITIVE
Proline	FALSE	116.0706	70.1	3	380	17	POSITIVE
Putrescine	FALSE	89.1073	72.1	3	380	8	POSITIVE
Quinic acid	FALSE	193.0707	111.04	3	380	10	POSITIVE
SAH	FALSE	385.1289	136.1	3	380	33	POSITIVE
Sarcosine/Alainine	FALSE	90.055	44.1	3	380	20	POSITIVE
Sarcosine/Alanine	FALSE	90.055	72.1	3	380	9	POSITIVE
Serine	FALSE	106.0499	60.1	3	380	9	POSITIVE
Spermidine	FALSE	146.1652	72.1	3	380	17	POSITIVE
thiamine	FALSE	266.1196	122	3	380	19	POSITIVE
Thymine-d4	FALSE	131	113.9	3	380	16	POSITIVE
Tyrosine	FALSE	182.0812	136.1	3	380	9	POSITIVE
Uracil	FALSE	113	72	3	380	4	POSITIVE
Urea	FALSE	61.0396	44	3	380	10	POSITIVE
Valine	FALSE	118.0863	72.2	3	380	9	POSITIVE
Xanthine	FALSE	153.04	110	3	380	21	POSITIVE
xanthosine-5-phosphate	FALSE	365.0493	97	3	380	13	POSITIVE

Disease Models & Mechanisms • Supplementary information

Table S5. 49 Detected metabolites and Multiple reaction monitoring (MRM) method of Yeast samples analysed in negative ionization mode.

Compound Name	ISD	Precursor ion	Product ion	Dwell	Frag(V)	CE(V)	Polarity
1,3-diphopshateglycerate	FALSE	265	79	3	380	37	NEGATIVE
2,3-dihydroxybenzoic acid	FALSE	153	109	3	380	19	NEGATIVE
3-phosphoglycerate	FALSE	185	97	3	380	17	NEGATIVE
6-phospho-D-gluconate	FALSE	275	97	3	380	13	NEGATIVE
allantoin	FALSE	157.05	114	3	380	17	NEGATIVE
anthranilate	FALSE	136	92	3	380	18	NEGATIVE
dihydroorotate	FALSE	157	113	3	380	14	NEGATIVE
D-sedoheptulose-1-7-phosphate	FALSE	289	97	3	380	27	NEGATIVE
Eicasatetraenoic acid-D8 (ETA)	FALSE	311.5	59.05	3	380	35	NEGATIVE
Farnesyl-PP	FALSE	381	78.7	3	380	40	NEGATIVE
FBP/GBP	FALSE	338.9	79	3	380	60	NEGATIVE
Fumarate	FALSE	119	74	3	380	5	NEGATIVE
Fumaric acid	FALSE	115	71.1	3	380	5	NEGATIVE
Glucose-6-Phosphate	FALSE	259	97	3	380	5	NEGATIVE
Glutamic acid D5	FALSE	151	107	3	380	13	NEGATIVE
Glutamic acid-D5	FALSE	151	107	3	380	5	NEGATIVE
Glutamine	FALSE	145	109	3	380	9	NEGATIVE
HBA	FALSE	103	59.1	3	380	5	NEGATIVE
hexose-phosphate	FALSE	259	79	3	380	42	NEGATIVE
Hydroxyisocaproic acid	FALSE	131.006	85.1	3	380	16	NEGATIVE
inosine	FALSE	267	135	3	380	27	NEGATIVE
Ketoglutarate	FALSE	145	101.1	3	380	5	NEGATIVE
lactate	FALSE	89	43.2	3	380	16	NEGATIVE
L-Anthranilic aicd	FALSE	137	93.1	3	380	15	NEGATIVE
L-Glutamic acid D5	FALSE	151	107	3	380	13	NEGATIVE
L-Tryptophan	TRUE	205	117	3	380	10	NEGATIVE
Malic acid	FALSE	133	115.1	3	380	5	NEGATIVE
Malonate (Propanedioate)	FALSE	103.1	59.1	3	380	5	NEGATIVE
Maltose	FALSE	341.1	161	3	380	5	NEGATIVE
MALTOTETRAOSE	FALSE	665.2	161.1	3	380	13	NEGATIVE
N-acety AA-d3	FALSE	177	90.8	3	380	6	NEGATIVE
N-Aetyl Aspartic acid	FALSE	174	88.1	3	380	13	NEGATIVE
octulose-monophosphate (O8P-O1P)	FALSE	319	97	3	380	22	NEGATIVE
p-aminobenzoate	FALSE	136.05	92	3	380	18	NEGATIVE
PEP	FALSE	167	79	3	380	9	NEGATIVE
Ribose-5-phosphate	FALSE	229.01	96.97	3	380	15	NEGATIVE
shikimate	FALSE	173	93	3	380	20	NEGATIVE
sn-glycerol-3-phosphate	FALSE	171	79	3	380	15	NEGATIVE
succinate	FALSE	117	73	3	380	12	NEGATIVE
taurine	FALSE	124	80	3	380	18	NEGATIVE
Tetradecanoic acid-D3 (TDA-D3)	FALSE	230.3	59	3	380	40	NEGATIVE
trehalose-6-Phosphate	FALSE	421	79	3	380	36	NEGATIVE
UDP-D-glucose	FALSE	565	323	3	380	25	NEGATIVE
UDP-N-acetyl-glucosamine	FALSE	606	385	3	380	28	NEGATIVE
Uric acid	FALSE	167.001	124	3	380	17	NEGATIVE
ADP	FALSE	426.1	159	3	380	27	NEGATIVE
dGDP	FALSE	426.12	159	3	380	27	NEGATIVE
Frctose-6-Phosphate	FALSE	259	79.1	3	380	37	NEGATIVE
NAD+	FALSE	662	540	3	380	21	NEGATIVE

Disease Models & Mechanisms • Supplementary information

Table S6. LC-MS system run parameters.

Source Parameters			
**Parameter**	**Value (+)**	**Value (-)**	
Gas Temp (°C)	250	250	
Gas Flow (l/min)	14	14	
Nebulizer (psi)	20	20	
SheathGasHeater	350	350	
SheathGasFlow	12	12	
Capillary (V)	3000	3000	
VCharging	1000	1000	
**Ion Funnel Parameters**			
Pos High Pressure RF	150		
Pos Low Pressure RF	60		
Neg High Pressure RF	150		
Neg Low Pressure RF	60		
**Auxiliary**			
Draw Speed	100.0 µL/min		
Eject Speed	100.0 µL/min		
Draw Position Offset	0.0 mm		
Wait Time After Drawing	2.0 s		
Vial/Well bottom sensing	Yes		
**Injection**			
Injection Mode	Injection with needle wash		
Injection Volume	2.00 µL		
**Needle Wash**			
Needle Wash Location	Flush Port		
Wash Time	10.0 s		
Flow	0.300 mL/min		
Use Solvent Types	Yes		
Stroke Mode	Synchronized		
Low Pressure Limit	0.00 bar		
High Pressure Limit	600.00 bar		
Max. Flow Ramp Up	100.000 mL/min²		
Max. Flow Ramp Down	100.000 mL/min²		
Expected Mixer	No check		
**Stop Time**			
Stoptime Mode	Time set		
Stoptime	23.00 min		
Post time mode	Time set		
Post time	5.00min		
**Column Mode**	**Solvent A**	**Solvent B**	
Water's Positive Mode	Grade water (Water, Optima™ LC/MS Grade, Cat. No. W6500, Fisher Chemical™, Fair Lawn, NJ, USA) + 0.1% formic acid (FA) (Formic Acid, 99.0 + %, Optima LC/MS grade, Cat. No. A117-50, Fisher Chemical, Fisher Scientific, Fair Lawn, NJ, USA)	100% Acetonitrile (ACN) (Acetonitrile, Optima LC/MS grade, Cat. No. A955, Fisher Chemical, Fisher Scientific, Fair Lawn, NJ, USA) + 0.1% formic acid (FA)	
Water's Negative Mode	95% Grade water (Water, Optima™ LC/MS Grade, Cat. No. W6500, Fisher Chemical™, Fair Lawn, NJ, USA) + 5% Acetonitrile + 20 mM Ammonium acetate (Ammonium Acetate (Optima LC/MS), Cat. No. A11450 Fisher Chemical, Fisher Scientific, Fair Lawn, NJ, USA), pH 9.0	100% Acetonitrile (ACN) (Acetonitrile, Optima LC/MS grade, Cat. No. A955, Fisher Chemical, Fisher Scientific, Fair Lawn, NJ, USA)	
**Timetable**			
**Time**	**Function**	**Parameter**	**Solvent composition**
1	3.00 min	Change Solvent Composition	Solvent Composition A:70.00% B:30.00 %
2	3.00 min	Change Flow	Flow: 0.3 mL/min
3	12.00 min	Change Solvent Composition	Solvent Composition A:98.00% B:2.00 %
4	12.00 min	Change Flow	Flow: 0.3 mL/min
5	15.00 min	Change Solvent Composition	Solvent Composition A:98.00% B:2.00 %
6	15.00 min	Change Flow	Flow: 0.3 mL/min
7	16.00 min	Change Solvent Composition	Solvent Composition A:15.00% B:85.00 %
8	16.00 min	Change Flow	Flow: 0.3 mL/min
9	23.00 min	Change Solvent Composition	Solvent Composition A:15.00% B:85.00 %
10	23.00 min	Change Flow	Flow: 0.3 mL/min
